# Niche analyses and the potential distribution of four invasive bumblebees worldwide

**DOI:** 10.1002/ece3.11200

**Published:** 2024-04-01

**Authors:** Tania Paola López‐Aguilar, Jose Montalva, Bruno Vilela, Marina P. Arbetman, Marcelo A. Aizen, Carolina L. Morales, Daniel de Paiva Silva

**Affiliations:** ^1^ Graduate Program in Natural Resources of the Cerrado State University of Goiás Anápolis Brazil; ^2^ Department of Biology National Autonomous University of Honduras in the Sula Valley (UNAH‐VS) San Pedro Sula Honduras; ^3^ Department of Biological and Environmental Sciences East Central University Ada Oklahoma USA; ^4^ Institute of Biology Federal University of Bahia Salvador Brazil; ^5^ Grupo de Ecología de la Polinización (EcoPol) INIBIOMA (CONICET, Universidad Nacional del Comahue) San Carlos de Bariloche Argentina; ^6^ Departamento de Ciências Biológicas, Conservation Biogegraphy and Macroecology Laboratory (COBIMA LAB) Instituto Federal Goiano Urutaí Brazil

**Keywords:** biodiversity loss, ecological niche, efficient pollinators, invasive bumblebees, potential distribution

## Abstract

The introduction of bees for agricultural production in distinct parts of the world and poor management have led to invasion processes that affect biodiversity, significantly impacting native species. Different *Bombus* species with invasive potential have been recorded spreading in different regions worldwide, generating ecological and economic losses. We applied environmental niche and potential distribution analyses to four species of the genus *Bombus* to evaluate the similarities and differences between their native and invaded ranges. We found that *B. impatiens* has an extended environmental niche, going from dry environmental conditions in the native range to warmer and wetter conditions in the invaded range. *Bombus ruderatus* also exhibited an extended environmental niche with drier and warmer conditions in the invaded range than in its native range. *Bombus subterraneus* expanded its environmental niche from cooler and wetter conditions in the native range to drier and warmer conditions in the invaded range. Finally, *B. terrestris* showed the most significant variation in the environmental niche, extending to areas with similar and different environmental conditions from its native range. The distribution models agreed with the known distributions for the four *Bombus* species, presenting geographic areas known to be occupied by each species in different regions worldwide. The niche analysis indicate shifts in the niches from the native to the invaded distribution area of the bee species. Still, niche similarities were observed in the areas of greatest suitability in the potential distribution for *B. ruderatus*, *B. subterraneus*, and *B. terrestris*, and to a lesser degree in the same areas with *B. impatiens*. These species require similar environmental conditions as in their native ranges to be established in their introduced ranges. Still, they can adapt to changes in temperature and humidity, allowing them to expand their ranges into new climatic conditions.

## Graphical abstract

The bees require similar conditions in their native and invaded ranges. The distribution models of the bees agreed with their known distributions. The bees shifted their niches during the invasion process.

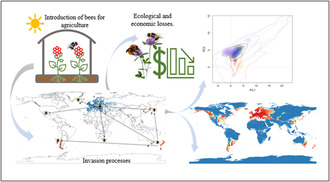

## 1 INTRODUCTION

Currently, the introduction of alien species is a topic of great interest in conservation biology due to their ecological impact on ecosystem functioning (Russo, 2016; Schmid‐Hempel et al., 2007; Valido et al., 2014), becoming a significant threat to global biodiversity and humanity (Acosta et al., 2016; IPBES, 2023; Russo, 2016). These species are defined as invasive when they exhibit tolerance to physicochemical conditions (Schmid‐Hempel et al., 2007), high reproductive capacity, the rapid expansion of their invaded distribution range after introduction (Acosta et al., 2016), and absence of natural enemies and competitors that can influence the size of their populations (Dafni et al., 2010). Furthermore, such characteristics allow them to modify the structure and dynamics of ecosystems (Aizen et al., 2008; Dafni et al., 2010), effectively integrating themselves into food chains, competing for different resources, and threatening native species (Iwasaki & Hoogendoorn, 2022; Schmid‐Hempel et al., 2007; Valido et al., 2014). Consequently, the ecological and economic effects of invasive alien species can be significant over time (Diagne et al., 2021; Ings et al., 2010; Pimentel et al., 2001, 2005). Specifically, the economic damages estimated to be caused by biological invasions have been estimated to have exceeded 100 billion dollars over 35 years, with an estimated annual expenditure of around 3 billion dollars (Adelino et al., 2021; Diagne et al., 2021; IPBES, 2023).

In different countries, the introduction of bumblebee species for agricultural pollination has been justified by their pollination efficiency or because of the ability of some to produce metabolic heat, allowing those capable of doing this to adapt to different climate types (Dafni et al., 2010; Montalva et al., 2017; Morales, 2007; Pérez, 2013; Winter & Adams, 2006). As a result, the number of invasion events by different invasive species is constantly increasing (Pérez, 2013). Still, the lack of information about the geographical distribution of species in introduced environments, the so‐called Wallacean deficit, causes setbacks that hinder conservation actions (Bini et al., 2006; Whittaker et al., 2005).

Therefore, developing and applying new methods and tools is needed to predict the areas most susceptible to species invasion (Acosta et al., 2016). Invasion of alien species is one of the direct drivers of change in nature with the largest global impact, along with changes in land and sea use, direct exploitation of organisms, pollution, and climate change (IPBES, 2019). Moreover, the spread and impact of invasive species are expected to amplify under global climate change in the near future (IPBES, 2023).

Considering the copious availability of species occurrences (Graham et al., 2004) and climatic data (Fick & Hijmans, 2017; Hijmans et al., 2005), new computational methods and approaches have been developed to comprehend species distribution and invasion ecology processes at broad scales. Species distribution models (SDM) have been applied to predict potential species invasion areas in the present and future (Andrade et al., 2019; Giannini et al., 2012; Peterson & Soberón, 2012; Silva et al., 2013, 2016). Additionally, macroecological methods have been used to better understand the potential evolutionary niche changes that invasive species may undergo during their invasion process (Broennimann et al., 2012; Guisan et al., 2014) and their potential impacts on native biota (Abrahamovich et al., 2007; Goulson, 2003; Pérez, 2013; Winter & Adams, 2006).

The bumblebee species *Bombus ruderatus* Fabricius, 1775, *Bombus subterraneus* Linnaeus, 1758, and *Bombus terrestris* Linnaeus, 1758, which originally had a Palearctic distribution, were introduced into New Zealand at the end of the 19th century (Goulson & Hanley, 2004). These introductions were more “artisanal,” with small numbers of fertilized queens. Still, after a series of introduction events in different countries (Pérez & Macías‐Hernández, 2012; Velthuis & Van Doorn, 2006), these species now occupy new ecoregions across the world (Abrahamovich & Díaz, 2002). In the decade of 1980, the rearing of *Bombus* species became commercial, with *B. terrestris* as the leading species of the bumblebee colony industry and trade (Dafni et al., 2010; Velthuis & Van Doorn, 2006). The commercialization worldwide and the increased number of initial propagules could significantly impact the invasion process (Simberloff, 2009). Similarly, *B. impatiens* Cresson, 1863, a species native to the Nearctics, became a commercial species in North America (Looney et al., 2019). *Bombus impatiens* currently extends into the Neotropics (Frison, 1926; Milliron, 1971). In addition to novel ecological contexts, these species may face evolutionary processes under their current invasion process, increasing their dispersal potential in new areas, modifying the ecosystem, and benefiting from interactions with native species in these new places.

Thus, in this work, we aimed to characterize the potential shifts in the environmental niche of these four bee species of *Bombus* from their native range to their invasion zones during their invasion processes. Specifically, we aim to examine the degree of divergence of their niches, comparing native and invaded ranges considering the current climate conditions for each species by conducting environmental analyses, ecological niche models, and potential distribution models under current climate conditions.

## 2 METHODS

### 2.1 Invasive species

**FIGURE 1:**
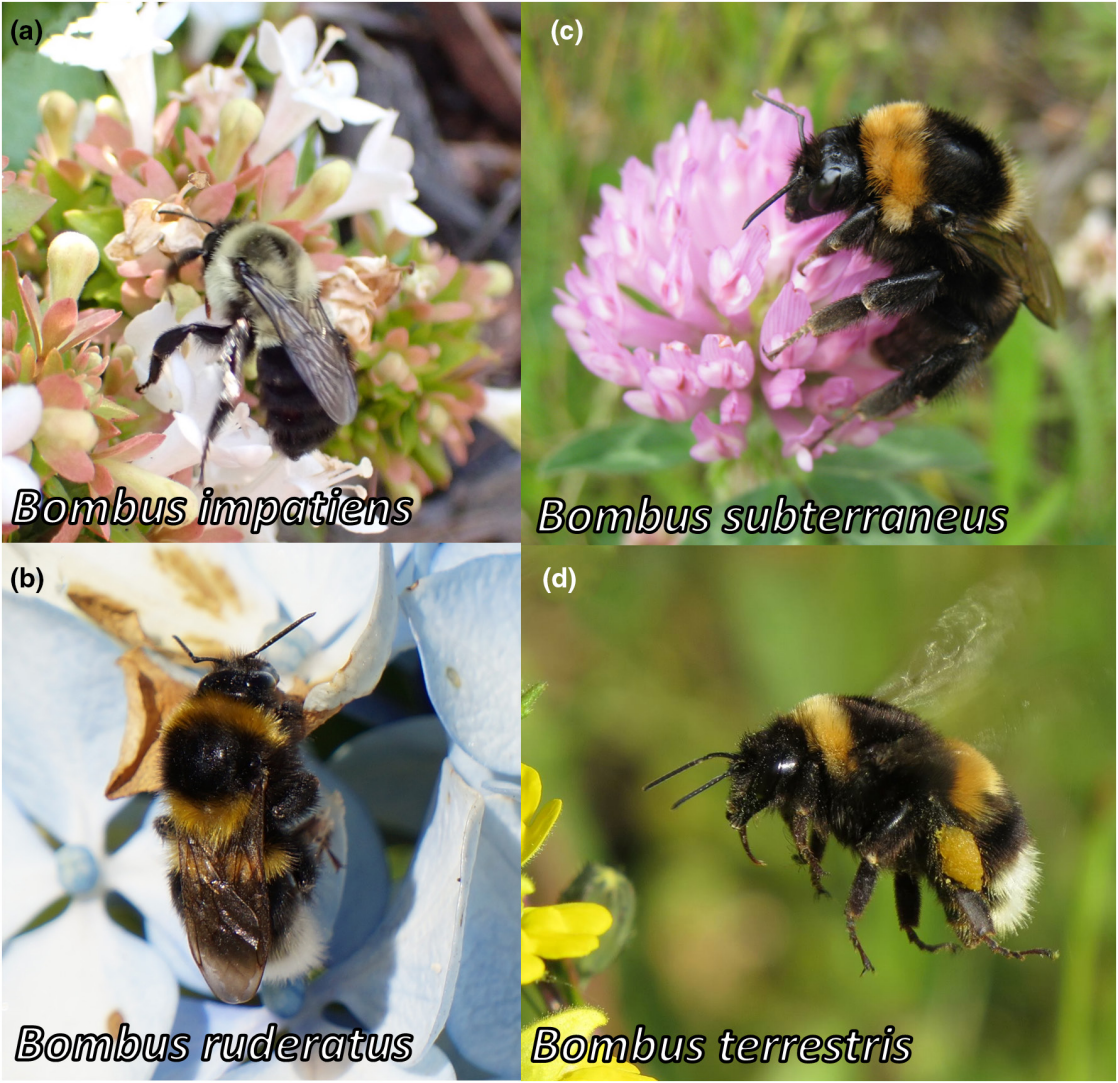
Bumblebee species analyzed in this study. (a) *Bombus impatiens*, (b) *B. ruderatus*, (c) *Bombus subterraneus*, and (d) *Bombus terrestris*. The photographs were courtesies from José Montalva, Fernando Tellez, Nikki Gammans, and Ninoska Pinto.

**FIGURE 2:**
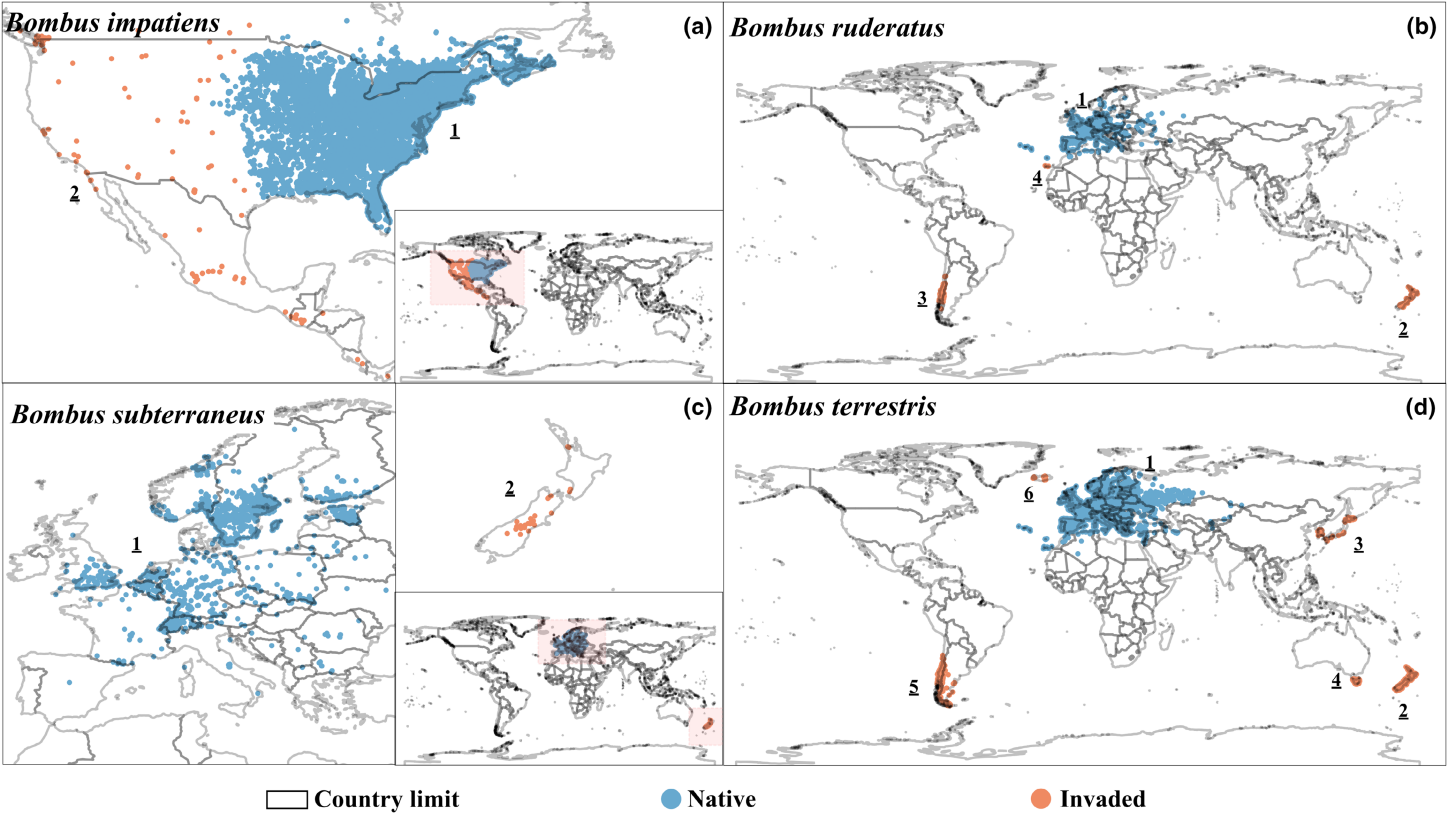
Occurrence maps of the *Bombus* species used in this study indicate their native and invaded distribution areas worldwide. (a) *Bombus impatiens*; native distribution: 1 Eastern United States of America and Canada (Nat); invaded distribution: 2 Western United States of America, Canada, and Mexico (Naw), and Central America (CA). (b) *Bombus. ruderatus*; native distribution: 1 Europe (Nat); invaded distribution: 2 New Zealand (NZ), 3 Chile and Argentina (SA), 4 Canary Islands (Can). (c) *Bombus subterraneus*; native range: 1 Europe (Nat); invaded range: 2 New Zealand (NZ). (d) *Bombus terrestris*; native range: 1 Europe, Western Asia, and Northern Africa (Nat); invaded range: 2 New Zealand (NZ), 3: Japan and South Korea (ASe), 4 Tasmania (Tas), 5 Chile and Argentina (SA), 6 Iceland (ISL).

Ten *Bombus* species have been recorded to invade new ranges worldwide from their native areas (Table 1; Hallmen, 2017; Russo, 2016). These species have been introduced into different countries for their efficiency in agricultural pollination (Dafni et al., 2010; Goulson, 2003; Sachman‐Ruiz et al., 2015). For this study, we selected four of the most widespread invasive species of the genus (Velthuis & Van Doorn, 2006) that are still commercialized and present more reliable identity and invasion data. These species are *B. impatiens* (Figures 1a and 2a), *B. ruderatus* (Figures 1b and 2b), *B. subterraneus* (Figures 1c and 2c), and *B. terrestris* (Figures 1d and 2d).

**TABLE 1:** *Bombus* species reported to be invasive. The names of the species, range classes, and the year of introduction are provided. For species with two different dates, the first relates to “artisanal” introductions, when a few fertilized queens/colonies were introduced. In contrast, the second date regards the “commercial” introduction of the species, when thousands of queens/colonies were introduced in the invaded ranges.

Species		Year	From	Found in	References
*Bombus hortorum*	Introduced	1885	UK	New Zealand	Goulson and Hanley (2004), Hallmen (2017), Russo (2016)
	Accidentally introduced	1950s	Europe	Iceland	
*Bombus hypnorum*	Naturally expanding or shifting range	2001	Europe	UK and Iceland	Goulson and Williams (2001), Hallmen (2017), Betts (2015), Russo (2016)
*Bombus impatiens*	Introduced	2003	North America (east)	Chile, Mexico, Central America, Canada	Ruz (2002), Ratti and Colla (2010), Looney et al. (2019), Russo (2016)
*Bombus jonellus*	Accidentally introduced	900s	Europe	Iceland	Hallmen (2017)
*Bombus lucorum*	Naturally expanding or shifting range	1979	Europe	Iceland	Goulson (2003), Hallmen (2017), Russo (2016)
*Bombus pascuorum*	Naturally expanding or shifting range	2010	Europe	Iceland	Hallmen (2017)
*Bombus pratorum*	Naturally expanding or shifting range	2010	Europe	Iceland	Hallmen (2017)
*Bombus ruderatus*	Introduced	1885	UK	New Zealand, Chile, Argentina, Patagonia, Canary Islands	Ruz (2002), Goulson and Hanley (2004), Montalva et al. (2011), Russo (2016)
		1983			
*Bombus subterraneus*	Introduced	1885	UK	New Zealand	Goulson and Hanley (2004), Russo (2016)
*Bombus terrestris*	Introduced	1885	UK, Europe	Chile, China, Israel, Japan, Mexico, South Africa, South Korea, New Zealand, Tasmania, and Taiwan	Ruz (2002), Goulson and Hanley (2004), Dafni et al. (2010); Montalva et al. (2011), Russo (2016), Naeem et al. (2018)
		1990s			

### 2.2 Occurrence data of the analyzed species

*Bombus impatiens* is a species native to the eastern United States of America and Canada (Nat designation in all analyses hereon) (Figure 2a1). However, in 2003 (Russo, 2016), this species was introduced in the western United States of America and Canada, as well as in Mexico (Figure 2a2, Naw designation in all analyses hereon) and Central America (Figure 2a2, CA designation in all analyses hereon) (Morales, 2007; Russo, 2016), for the pollination of different crops, such as blueberries, tomatoes, and peppers (Sachman‐Ruiz et al., 2015).

*Bombus ruderatus* is native to Eurasia (Nat designation in all analyses hereon) (Figure 2b1). In 1883, it was introduced into New Zealand (NZ) (Figure 2b2) for the pollination of clover (*Trifolium pratense*) (Goulson & Hanley, 2004). From NZ, the queens were collected in the wild and then released into Chile (SA designation in all analyses hereon) (Figure 2b3) (Abrahamovich & Díaz, 2002; Arretz & Macfarlane, 1986; Montalva, 2012). Currently, this species has been recorded with widespread and stable populations in Argentina for more than 30 years (Figure 2b3) (Lohrmann et al., 2022; Madjidian et al., 2008; Morales, 2007; Morales et al., 2013) and the Canary Islands (Can designation in all analyses hereon) (Figure 2b4) (Pérez & Macías‐Hernández, 2012; Russo, 2016).

*Bombus subterraneus* is a native species of Europe (Nat designation in all analyses hereon) (Figure 2c1) (Aase et al., 2011; Williams, 1982). However, in 1885, it was introduced in New Zealand (NZ designation in all analyses hereon) (Morales, 2007; Russo, 2016; Stewart et al., 2010; Figure 2c2) for clover pollination (Goulson & Hanley, 2004).

*Bombus terrestris* is a species native to Europe, North Africa, and West Asia (Nat designation in all analyses hereon) (Figure 2d1) (Rasmont et al., 2008). It was first translocated outside its native range in 1881 when it was introduced to New Zealand by European settlers to improve crop pollination, particularly of some fodder crops like clovers (NZ) (Figure 2d2) (Dafni et al., 2010; Goulson & Hanley, 2004). However, the boom of *B. terrestris* translocation began with its industrial rearing and global trade starting in the 1980s. Since then, commercial colonies of this species have been introduced to Israel, Japan, South Korea (ASe designation in all analyses hereon) (Figure 2d3), Chile (SA designation in all analyses hereon; Figure 2d5), and Iceland (Figure 2d6) (Hallmen, 2017), managing to spread to Argentina, Tasmania (Tas designation in all analyses hereon) (Figure 2d4), and other countries (Acosta et al., 2016; Goulson, 2003; Montalva et al., 2011; Morales, 2007; Russo, 2016).

### 2.3 Occurrence data

Occurrence records for the four *Bombus* species were obtained from online databases: The Global Biological Information Facility and related institutions (GBIF; http://www.gbif.org; *B. terrestris*: 10.15468/dl.255jus; *B. ruderatus*: 10.15468/dl.xw5yaz; *B. subterraneus*: 10.15468/dl.8u4zyt; *B. impatiens* 10.15468/dl.4vjt5p), photo‐confirmed identifications in BugGuide.net (http://www.bugguide.net), confirmed records from iNaturalist.org (https://www.inaturalist.org/; acknowledgments to the taxonomic specialists are available in the end of the manuscript), and National Biodiversity Network (NBN Atlas; https://nbnatlas.org/) (Acosta, 2015). In addition, Google Earth: 7.3.2.549123/2018 and Global Gazetteer version 2.3 (http://www.fallingrain.com/world/) were used to obtain the proxy coordinates of confirmed photographic records and museum records that indicated locality but lacked geographic coordinates.

The occurrence records were managed in a matrix for each species with scientific name, latitude, longitude, and distribution data classified as native or invaded. These were later divided into the regional designations mentioned above for all four analyzed species. Subsequently, these data were manually filtered, eliminating duplicate occurrence values and those with errors (doubtful records, in the ocean, and empty values) and were validated by experts, gathering 122,257 occurrences for *B. impatiens*, 2950 occurrences for *B. ruderatus*, 5174 occurrences for *B. subterraneus*, and 76,246 occurrences for *B. terrestris*.

We applied a minimum 5 km radius buffer of the distance between each point to minimize geographic information biases using the R package spThin (Aiello‐Lammens et al., 2015), resulting in 4535 occurrences for *B. impatiens*, 1032 for *B. ruderatus*, 1023 for *B. subterraneus*, and 6195 for *B. terrestris*.

### 2.4 Environmental data

The current climate dataset of the 19 temperature and precipitation bioclimatic variables was obtained from WorldClim 2.1 with a spatial resolution of 5 arc‐min (10 km × 10 km pixel) (http://www.worldclim.org) (Fick & Hijmans, 2017; Hijmans et al., 2005). These variables comprise the 1970–2000 temporal range and are often used in macroecological analyses involving invasive species.

### 2.5 Niche analysis

We assessed the environmental niche for each species by applying the method developed by Broennimann et al. (2012) to determine whether the niche of the four species has changed since dispersal from native to invaded areas. First, we defined geographic backgrounds as a buffer of one‐degree width around a minimum convex polygon based on the occurrence records of each species in each region (Silva et al., 2016; Teles et al., 2022). This buffer was used to delimit the area that can be geographically accessible by dispersal for each species. Next, we applied PCA‐env, simultaneously considering the space occupied by each species and contrasting the environmental conditions of the native distribution areas with the invaded distribution areas (Broennimann et al., 2012; Silva et al., 2016; Teles et al., 2022). Then, we calculated Schoener's D metric (Broennimann et al., 2012) for niche overlap, with values ranging between 0 (no overlap) and 1 (complete overlap). We applied the similarity test with 100 interactions to compare whether the niche of the native area significantly overlaps with the niche of the invaded area in each species. Subsequently, we calculated the metrics of niche unfilling, niche stability, and niche expansion, following the analyses proposed by Guisan et al. (2014). Niche unfilling refers to the proportion of the native niche not occupied by the species in the invaded areas. Niche stability measures the proportion of the invaded niche that remained stable compared to the analogous climate available in the native niche. In contrast, niche expansion indicates new environmental conditions occupied by the invaded population (the sum between niche stability and expansion should be 1). These ratios ranged from 0 to 1 and were calculated by contrasting each species native area with their corresponding invaded area.

### 2.6 Modeling process

We applied the method “CELLSIZE” based on the spThin package of R to have an equal size in the grid of cells (Aiello‐Lammens et al., 2015), and obtain the data of unique geographical occurrences for each species, considering the distance of thinning in the analysis (Andrade et al., 2020). The standardized data of the 19 bioclimatic variables for the current scenario were subjected to a principal component analysis (PCA). We then selected the first six resulting principal components (PC) (considering a 95% explanation of the original climatic variation) as predictors of the ranges of the species to reduce collinearity and select the model variables. Before we ran the PCA analysis, the variables were z‐transformed. In this method, the mean value of each variable is subtracted from the raw value of all grid cells from each variable, and the resulting value is then divided by the standard deviation of the variable. With this transformation, the mean value of each variable equals zero, and the highest positive/negative values of each variable are always equal to |1|. In addition, pseudo‐absence points were generated by applying the “environmental‐constrain” method, in which pseudo‐absences were allocated to areas of low environmental suitability predicted by a bioclimatic model (Andrade et al., 2020; Barbet‐Massin et al., 2012). Finally, the fit and evaluation of the model were determined by the structured geographic partitioning method, applying the “BLOCK” method (Andrade et al., 2020).

We used the following algorithms to build the models: (1) Maximum entropy (MAXENT), with the linear and quadratic functions that have been defined by default (Phillips et al., 2006), (2) support vector machines (SVM), which is a robust prediction method (Salcedo‐Sanz et al., 2014), (3) random forest (RDF) (Liaw & Wiener, 2002), and (4) Boosted regression tree (BRT) (Friedman, 2001), which provides a prediction model in the form of decision trees (Andrade et al., 2020). Our primary motivation for selecting this set of algorithms lies in their well‐demonstrated effectiveness in capturing complex relationships in ecological data and, therefore, having a higher performance in predicting geographic distributions in most scenarios when compared to distance‐based or regression algorithms (e.g., Valavi et al., 2022). We used the Jaccard (Leroy et al., 2018) index to evaluate the climatic similarity in the datasets. We selected the posterior spatial constraint based on the occurrence constraint (Andrade et al., 2020).

### 2.7 Maps

The maps were built using QGIS 3.20 (QGIS Development Team, 2021). The occupancy map was edited from the species occurrence points, using the colorblind safe palette with 3‐class RdBu from ColorBrewer 2.0 (https://colorbrewer2.org) and taking as reference the world boundary map, obtained in DIVA‐GIS (https://www.diva‐gis.org/). The maps of the potential distribution models were reclassified with the colorblind color gradient with five classes. All models were run with the ENMTML package of Andrade et al. (2020) and, together with all analyses, were processed in R 4.2.1. environment (R Core Team, 2022).

## 3 RESULTS

### 3.1 Niche analysis

In the multivariate analyses performed, the first two axes of the PCA jointly explained 72%–75% of the environmental variation in the four species (Table 2, Figures S1A, S2A, S3A, and S4A). The most important variables in the density plots for the four species were mean annual temperature (Bio1), minimum temperature of the coldest month (Bio6), mean temperature of the coldest quarter (Bio11) for the first principal component, mean temperature of warmest quarter (Bio10) and maximum temperature of the warmest month (Bio5) for the second principal component, and additionally in *B. terrestris* the mean temperature of the driest quarter (Bio9). The contributions of each variable on each axis of the PCA are presented in Figures S1B–C, S2B–C, S3B–C, and S4B–C.

**TABLE 2:** Percentages of environmental variation in niche analysis by the first two PCA axes for each *Bombus* species, along with the most important variables from each case.

Species	Variables (PCA1/PCA2)	PCA%	PC1%	PC2%
*B. impatiens*	Bio1, Bio6, Bio11/Bio5, Bio10	71.97	43.12	28.85
*B. ruderatus*	Bio1, Bio6, Bio11/Bio5, Bio10	72.52	41.05	31.47
*B. subterraneus*	Bio1, Bio6, Bio11/Bio5, Bio10	75.31	43.00	32.30
*B. terrestris*	Bio1, Bio6, Bio9, Bio11/Bio5, Bio10	74.88	44.30	30.57

^a^ The specific names for each one of the “Bio” variables are shown in Table 5.

**FIGURE 3:**
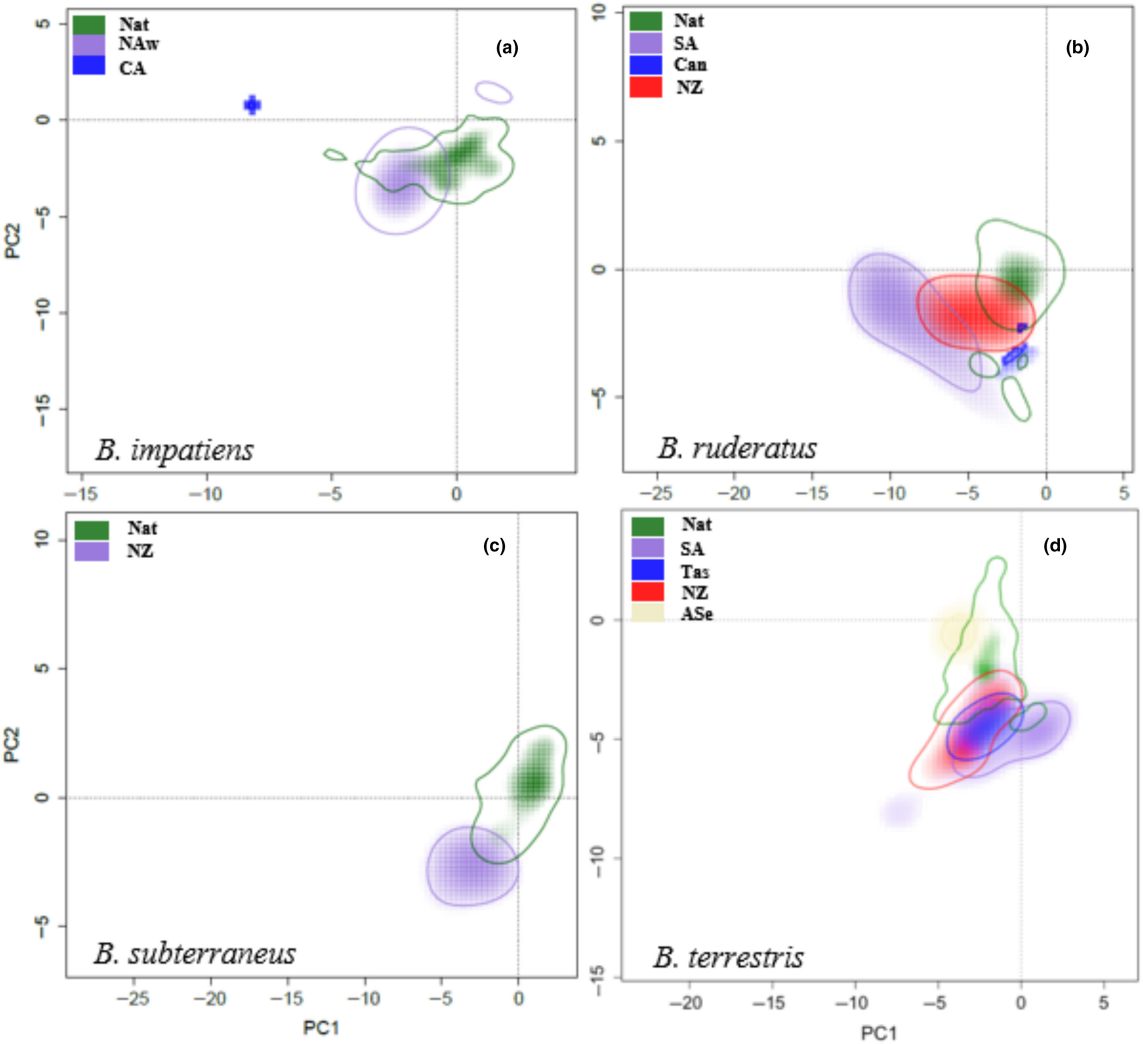
Overlap of the climatic niche of the *Bombus* species across native and invaded ranges. (a) *Bombus impatiens*: Native = Nat (green), invaded: Western North America = NAw (purple), Central America = CA (blue). (b) *Bombus ruderatus*: Native: Nat (green), Invaded: South America = SA (purple), Canary Islands: Can (blue), New Zealand = NZ (red). (c) *Bombus subterraneus*: Native Nat (green), Invaded: New Zealand: NZ (purple). (d) *Bombus terrestris*: Native: Nat (red), Invaded: New Zealand: NZ (green), South America: SA (purple), Tasmania: Tas (blue), East Asia: ASe (beige). The solid line represents 10% of the occurrence density.

In pairwise comparisons across all (native vs. invaded) distribution ranges for the four species, they showed variable proportions of overlap, ranging from 0.000 to 0.329 for *B. impatiens*, 0.000 to 0.125 for *B. ruderatus*, 0.149 for *B. subterraneus*, and 0.000 to 0.455 for *B. terrestris* (Table 3, Tables S1, S6, S11, and S16). When comparing the native area with each invaded area in each species, for *B. impatiens*, the NAw area showed overlaps of 0.329 and 0.000 in CA. The NAw area population occupies slightly drier and warmer climatic conditions than the native area. On the other hand, the CA population occupies wetter and warmer conditions than the native area (Figure 3a, Figure S5). For *B. ruderatus*, NZ showed a high overlap (0.125), followed by SA (0.057) and Can (0.002). Here, the population in Can occupies drier climatic conditions than the native area, NZ occupies slightly drier and warmer conditions than the native area, and SA occupies drier and warmer climatic conditions than the native range (Figure 3b, Figure S6). *Bombus subterraneus* has only an invaded area in NZ, which shows an overlap of (0.149) due to it occupying drier and warmer climatic conditions than in the native area (Figure 3c, Figure S7). In *B. terrestris*, the Tas area showed the highest proportion of overlap (0.398), followed by NZ (0.259), SA (0.119), and ASe (0. 101). In Tas and NZ, the populations occupied wetter climatic conditions than the native area. In ASe, they occupy similar conditions. In comparison, in SA, they occupied wetter climatic conditions and a greater range of temperatures compared with the native area. A comparison analysis was not performed for ILS due to the slight environmental variation and the few occurrence points (Figure 3d, Figure S8).

**TABLE 3:** Comparison of niche overlap (D), niche similarity, niche unfilling, niche stability, and niche expansion between native areas and each invaded area for each *Bombus* species, according to Broennimann et al. (2012).

Species	ERs	Overlap (D)	Similarity test native → ERs, *p*‐values	Niche unfilling native → ERs	Niche stability native → ERs	Niche expansion native → ERs
*B. impatiens*	NAw	0.329	**0.010**	0.274	0.857	0.143
	CA	0.000	1.000	1.000	0.000	1.000
*B. ruderatus*	SA	0.057	0.267	0.551	0.382	0.618
	Can	0.002	0.208	0.000	0.013	0.987
	NZ	0.125	**0** **.050**	0.196	0.764	0.236
*B. subterraneus*	NZ	0.149	**0.050**	0.740	0.530	0.470
*B. terrestris*	NZ	0.259	**0.020**	0.284	0.645	0.355
	SA	0.119	**0.040**	0.280	0.607	0.393
	Tas	0.398	**0.010**	0.672	0.797	0.203
	ASe	0.101	**0.050**	0.652	0.858	0.142

*Note*: The niche similarity test checks if the niche overlap between two areas is greater than randomly expected. Values in bold represent significant *p*‐values (*α* = .05).

Abbreviations: ASe, East Asia; CA, Central America; Can, Canary Islands; ERs, invaded ranges; NAw, Western North America; NZ, New Zealand; SA, South America; Tas, Tasmania.

In the similarity test, assuming an alpha value of 0.05 when contrasting the invaded areas with the native areas, *B. impatiens* showed a climatic niche in the NAw range that was significantly similar to the native range and showed no similarity in the CA range with the native range (Table 3, Table S2). *Bombus ruderatus* showed a significantly similar climatic niche in NZ and no similarity between AS and Can (Table 3, Table S7). *Bombus subterraneus* showed a significantly similar climatic niche in NZ (Table 3, Table S12). *Bombus terrestris* showed a similar climatic niche in all invaded range areas (Table 3, Table S17). Figure 3 illustrates the proportions of overlap among all the distribution areas considered in this analysis.

Finally, the climatic niches in the invaded areas presented for *B. impatiens*, a high degree of space occupancy of the original niche, high stability, and a small niche expansion in NAw, while there was no overlap in CA (i.e., maximum unfilling and expansion, and no stability) (Table 3, Tables S3–S5). For *B. ruderatus*, there was a medium degree of occupancy of the original niche, with more expansion than stability in SA, a high degree of occupancy in Can and NZ, with high expansion in Can and more stability in NZ (Table 3, Tables S8–S10). Regarding *B. subterraneus*, there was a low degree of occupancy with stability and expansion in NZ (Table 3, Tables S13–S15); and *B. terrestris*, a high degree of occupancy in NZ and SA and a medium degree of occupancy in Tas and ASe, and higher stability than expansion in all invaded areas (Table 3, Tables S18–S20). Even when most invaded areas in the distinct species showed significant niche similarity with the corresponding native areas, the dissimilarity occurred in the niche for CA in *B. impatiens*. SA and Can in *B. ruderatus* indicated a difference in the environmental characteristics of these regions compared to the native area (Tables S2 and S7).

### 3.2 The current distribution of the four *Bombus* species

**FIGURE 4:**
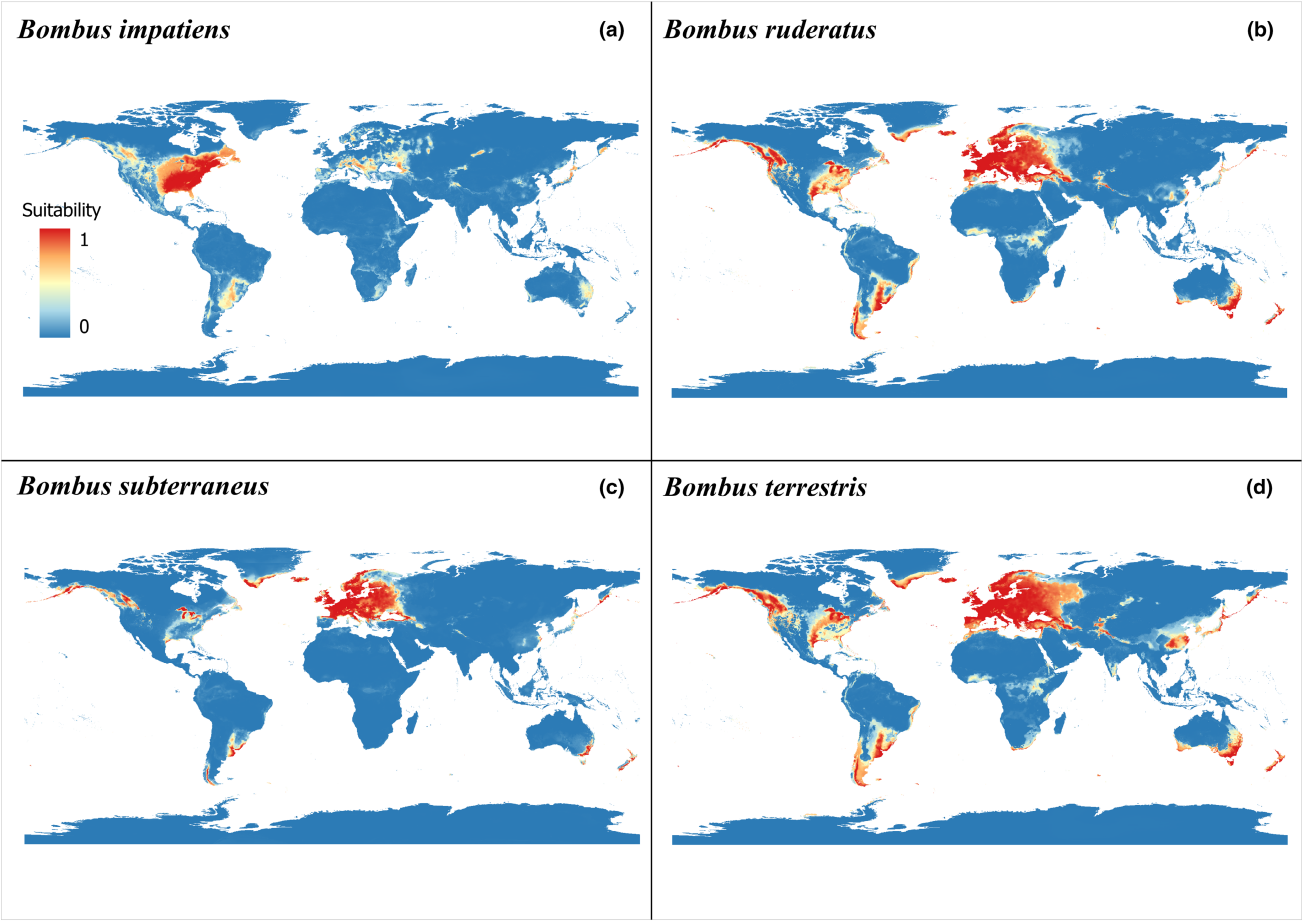
Map of the current potential distribution of *Bombus* species, indicating the distribution areas with major environmental suitability worldwide. (a) *Bombus impatiens*, (b) *Bombus ruderatus*, (c) *Bombus subterraneus*, and (d) *Bombus terrestris*.

The current potential distribution model showed congruence with the known distribution for the four *Bombus* species, presenting a geographic expansion with suitable environmental conditions for the occurrence of each one (Figure 4). The Jaccard values of the metrics used in evaluating all algorithms were >0.9, and the best predictor algorithms of the model were SVM and BRT. The weighted ensemble consensus method was used for this task (Table 4). The first three principal components explained the greatest environmental variation of the model in each species with 83% of the variation, where the variables with the greatest correlational significance are positive correlation (Bio1, Bio2, Bio4, Bio5, Bio6, Bio7, Bio9, Bio10, and Bio11), and negative correlation (Bio12, Bio14, Bio15, Bio17, and Bio19) (Table 5).

**TABLE 4:** Results of the evaluation of the algorithms used in constructing the distribution model.

Species	Algorithms				
	BRT	MXS	RDF	SVM	WMEA
*B. impatiens*	0.96	0.96	0.94	0.96	0.96
*B. ruderatus*	0.97	0.94	0.96	0.96	0.97
*B. subterraneus*	0.97	0.95	0.98	0.98	0.98
*B. terrestris*	0.97	0.93	0.97	0.97	0.97

Abbreviations: BRT, boosted regression trees; MXS, maxent; RDF, random forest; SVM, support vector machines; WMEA, ensemble prediction.

**TABLE 5:** Bioclimatic variables with high correlation in the potential distribution model.

Bioclimatic variables	PC1	PC2	PC3
Bio1: Annual mean temperature	**0.2784**	0.2150	0.0447
Bio10: Mean temperature of warmest quarter	0.2557	**0.2609**	0.1636
Bio11: Mean temperature of coldest quarter	**0.2876**	0.1644	−0.0680
Bio12: Annual precipitation	0.2512	**−0.2803**	0.0589
Bio14: Precipitation of driest month	0.1744	**−0.3176**	**0.2796**
Bio15: Precipitation seasonality (Coefficient of variation)	−0.1200	0.0221	**−0.4387**
Bio17: Precipitation of driest quarter	0.1824	**−0.3248**	**0.2658**
Bio19: Precipitation of coldest quarter	0.1796	**−0.3056**	0.0468
Bio2: Mean diurnal range (Mean of monthly (↑T° – ↓T°))	0.1207	**0.3699**	−0.0164
Bio4: Temperature seasonality (standard deviation ×100)	−0.2147	0.0831	**0.4441**
Bio5: Max temperature of warmest month	0.2493	**0.2799**	0.1686
Bio6: Min temperature of coldest month	**0.2892**	0.1442	−0.0693
Bio7: Temperature annual range (BIO5‐BIO6)	−0.1772	0.1884	**0.4486**
Bio9: Mean temperature of driest quarter	**0.2704**	0.1619	−0.1367

*Note*: Bold values indicate correlations with more significant effects (*r* > ±.25).

Jaccard values close to 1 indicate a good agreement between the predictions and the original occurrence data of the species. In the generated models, *B. impatiens* showed an extension of its potential distribution from the current area of occupancy northward into Canada and Alaska and, in low suitability, into southern Mexico and Central America. It also showed potential suitability regions in Europe, southern South America, small, isolated regions in Asia and Australia, and medium–low suitability in South Africa. For *B. ruderatus*, the potential distribution with high suitability conditions extends over areas of current occupancy. It showed regions of high suitability in North America, Australia, Iceland, and South Africa and small isolated strips in Asia. It also showed medium–low suitability strips in the central region of Africa. In *B. subterraneus*, the potential distribution showed suitable areas in southern South America, northern North America, Iceland, southeastern Australia, and small strips in eastern Asia and *B. terrestris*. Its potential distribution showed considerable expansion in the available range and a suitable region in North America and Africa.

## 4 DISCUSSION

To properly understand the different processes of biological invasions (Broennimann et al., 2007; Pearman et al., 2008), it is necessary to comprehend niche shift/conservatism (Silva et al., 2016) and analyze it through different spatiotemporal scales. Therefore, it will be possible to evaluate whether the niche of a species changes or remains conserved during the onset of the invasion process (Broennimann et al., 2007; Pearman et al., 2008). Our results show that the invasions in each *Bombus* species gave rise to significantly different realized niches. The variations in the environmental niche of the invaded areas with the native area for the four species are perceived through similarity and overlap analyses. In the potential distribution, it is observed that the three European species share potential areas of suitability, and the North American species are more restricted in environmental conditions. The adaptive capacity could influence this, and these species must expand the environmental niche beyond the climatic limits in native areas, as has occurred with other species (Broennimann et al., 2007; Silva et al., 2016).

Several factors could influence the observed expansions in the environmental niche beyond the climatic limits in native areas. One influential factor is the adaptive capacity of species (Broennimann et al., 2007; Silva et al., 2016), particularly for social insects where the phenotypic plasticity could be playing an adaptive role at molecular, individual, and colony levels (Manfredini et al., 2019). Concerning the genetic pool, many introduced species are subjected to population bottlenecks that erode genetic diversity and could limit their invasive potential. However, the continuous trade of *B. terrestris* to Chile has probably resulted in a high diversity mix due to the introduction of stocks from bumblebee factories located in several European countries and Israel, most likely rearing different subspecies of *B. terrestris* (Aizen et al., 2019). This condition allows the exploration of different niches and geographical expansion through the proliferation of novel genotypes via hybridization. Additionally and non‐exclusively, such expansions could result from a novel biotic context where the absence or reduction of natural predators or diverse bumblebee communities, as the ones occurring in Europe, allows invasive species to thrive and expand in their new environment (Keane & Crawley, 2002).

The differences in the climatic space occupied by these species are influenced by environmental variations that can be attributed to distinct factors (Broennimann et al., 2007), such as specific environmental conditions within different climatic zones (Novais, 2017) and physiological climatic tolerance limits of the species (Gouveia et al., 2014). These factors may affect behavior, physiology, reproduction, and species distribution, impacting the foraging areas and the ecosystem, which are important variables affecting these species (Gérard et al., 2022). However, the adaptations in some species of *Bombus*, such as the physiological mechanisms of thermoregulation and other key factors (Dafni et al., 2010; Wynants et al., 2021), together with the commercialization events in different countries and the poor management in the culture greenhouses, have potentiated the invasion processes in different regions of the world (Acosta, 2015; Sutherland et al., 2017), promoting potential mating with wild bees (Kubo et al., 2023; Miller et al., 2023; Naeem et al., 2018) and competition for resources with native species, to the degree of putting them at risk of extinction (Dafni et al., 2010; Esterio et al., 2013; Hallmen, 2017; Madjidian et al., 2008; Morales et al., 2013; Naeem et al., 2018; Pérez & Macías‐Hernández, 2012; Sachman‐Ruiz et al., 2015).

SDMs allow us to predict areas with potential conditions (Biella et al., 2017) for new invasion events (Broennimann et al., 2007; Naeem et al., 2018; Pearman et al., 2008; Teles et al., 2022). Here, the model generated for the four bumblebee species presented a positive correlation with temperature variables and a negative correlation with precipitation variables, whereby the latter could be one of the limiting factors in the occurrence of the species, as is the case with other temperature‐dependent congeners (Lu & Huang, 2023). Therefore, the potential cartographic representations show areas with suitable conditions that these species have not yet occupied. Consequently, they are expected not to become areas of invasion where these species have been commercialized and have not yet been registered as established (Dafni et al., 2010; Pérez & Macías‐Hernández, 2012; Velthuis & Van Doorn, 2006).

Our current potential distribution model for *B. terrestris*, which is the species that has been studied the most, agrees with previous predictions made at global (Acosta et al., 2016; Lecocq et al., 2016) and regional scales in South America (Acosta, 2015) and Asia (Naeem et al., 2018). Furthermore, our model suggests areas of potential suitability that show correspondences with already known invaded areas, extending to other countries in the southern cone of America, East Asia, and Australia, and also indicates potentially suitable areas in South Africa, Central Africa, the United States, Canada, Greenland, Caribbean Islands, and Honduras. In conjunction with the previous analysis, it can be said that this is a species tolerant to different conditions, so with these results, it is possible to foresee potential areas of invasion and prevent possible ecological risks in these areas related to poor marketing practices of domesticated colonies (Graystock et al., 2013; Schmid‐Hempel et al., 2014), the lack of conservation commitments by some countries (Aizen et al., 2019), and the adaptive capacities of the species (Dafni et al., 2010; Naeem et al., 2018; Morales, 2007; Smith‐Ramírez, et al., 2018; Velthuis & Van Doorn, 2006).

For *B. ruderatus*, *B. subterraneus*, and *B. impatiens*, no previous studies of potential distribution are known, so this would be the first global prediction of these species that, according to Dafni et al. (2010), Morales (2007), and Russo (2016), were introduced in different countries during different commercialization events. The *B. ruderatus* and *B. subterraneus* models agreed with the known invaded distributions. These also present potential distribution areas in common with *B. terrestris* because the three species are sympatric and require similar environmental conditions. For *B. ruderatus*, the greatest potential suitability is concentrated in North America, Australia, Tasmania, New Zealand, East Asia, Greenland, South Africa, and Iceland. This shows a possible expansion in the native and invasive range in South America. In *B. subterraneus*, the area of greatest potential suitability is concentrated in smaller traces in North America, South America, Iceland, Greenland, East Asia, New Zealand, and Australia, showing a possible expansion in the native area.

On the other hand, the distribution model of *B. impatiens* and the previous niche analysis in this work show that this species requires stricter environmental conditions, since the potential distribution is limited to small tracts of medium suitability in the invaded area in Europe, Australia, and East Asia. However, this does not diminish the potential ecological threats because this species is currently commercialized and (Iwasaki & Hoogendoorn, 2022; Morales, 2007; Torres‐Ruiz & Jones, 2012) interact with native species in the invaded area (Iwasaki & Hoogendoorn, 2022). Moreover, it has been documented that *B. impatiens* can hybridize with conspecifics (Morales, 2007; Naeem et al., 2018). Therefore, this species represents a threat to the Mesoamerican fauna that, although explained by another set of mechanisms, could have similar outcomes to *B. terrestris* in South America or Asia (Montalva et al., 2017; Morales et al., 2013; Naeem et al., 2018; Pérez, 2013).

## 5 CONCLUSION

Our results show that these bumblebee species tolerate different environmental conditions, which have allowed them to establish in new areas with different conditions from their native range and, consequently, to modify the fundamental niche during or after invasion processes.

The potential distribution of the distinct species coincides with places where some are present in invaded conditions under greenhouse management; therefore, if these species escape or are intentionally liberated, they can represent an invasion risk when they find favorable environmental conditions for their development. Consequently, it is of utmost importance to generate SDMs in different future scenarios to predict whether these species will continue to expand or contract their potential distribution and thus establish conservation measures for native species in possible invasion areas. It is also essential to evaluate other factors that may play a fundamental role in their distribution, so studies involving digital elevation models, land cover, plant–pollinator interactions, and comparing the interaction with native species are suggested to determine if there are niche overlaps.

## AUTHOR CONTRIBUTIONS

**Tania Paola López‐Aguilar:** Formal analysis (equal); investigation (equal); project administration (equal); validation (equal); visualization (equal); writing – original draft (equal); writing – review and editing (equal). **Jose Montalva:** Conceptualization (equal); data curation (equal); investigation (equal); validation (equal); writing – review and editing (equal). **Bruno Vilela:** Formal analysis (equal); methodology (equal); resources (equal); software (equal); visualization (equal); writing – review and editing (equal). **Marina P. Arbetman:** Conceptualization (equal); investigation (equal); writing – review and editing (equal). **Marcelo A. Aizen:** Conceptualization (equal); investigation (equal); writing – review and editing (equal). **Carolina L. Morales:** Conceptualization (equal); investigation (equal); writing – review and editing (equal). **Daniel de Paiva Silva:** Conceptualization (equal); investigation (equal); methodology (equal); project administration (equal); supervision (equal); visualization (equal); writing – original draft (equal); writing – review and editing (equal).

## CONFLICT OF INTEREST STATEMENT

The authors have no conflicts of interest to declare.

## Supporting information


Data S1.

## Data Availability

We confirm that the entire database used in this article is available in the Appendix and supplementary materials of this manuscript.
