# Supplementary material for: Niche analyses and the potential distribution of four invasive bumblebees worldwide
Source: Ecol Evol. 2024 Apr 1;14(4):e11200. doi: 10.1002/ece3.11200 (PMC10985363; doi:10.1002/ece3.11200)
Supplement: Supplementary file 1 — Data S1. [file ECE3-14-e11200-s001.docx]

# Supplementary Material

**Article: Niche analyses and the potential distribution of four invasive bumble bees worldwide**

This document records the codes used to generate the results of the manuscript: “The biological invasion of bumble bees under the context of global anthropic changes” by Tania Paola López-Aguilar, Jose Montalva, Bruno Vilela, Marina P. Arbetman, Marcelo A. Aizen, Carolina L. Morales, and Daniel de Paiva Silva; thus, allowing readers the possibility of reproducing the analyses and results presented.

##

## **Supplementary Tables**

**Table S1** – Pair-wise niche overlap comparisons (D) between each of the exotic and native ranges of *B. impatiens*.

| NAw | NA | NA | NA |
| --- | --- | --- | --- |
| Nat | 0.329 | NA | NA |
| CA | 0.000 | 0 | NA |

Nat: Eastern North America native range; NAw: Western North America exotic range; CA: Central American exotic range.

**Table S2** – Pair‐wise niche similarity comparisons (p‐values) between each of the exotic and native ranges of *B. impatiens*.

|  |  | 2→1 |  |  |
| --- | --- | --- | --- | --- |
|  |  | NAw | Nat | CA |
| 2→1 | NAw | NA | 0.01 | 1 |
|  | Nat | 0.01 | NA | 1 |
|  | CA | 1.00 | 1.00 | NA |

Nat: Eastern North America native range; NAw: Western North America exotic range; CA: Central American exotic range.

**Table S3** – Pair‐wise niche unfilling proportions between each of the exotic and native ranges of *B. impatiens*.

| 2→1 | | | | |
| --- | --- | --- | --- | --- |
|  |  | NAw | Nat | CA |
| 2→1 | NAw | NA | 0.143 | 1 |
|  | Nat | 0.274 | NA | 1 |
|  | CA | 1.000 | 1.000 | NA |

Nat: Eastern North America native range; NAw: Western North America exotic range; CA: Central American exotic range.

**Table S4** – Pair‐wise niche stability proportions between each of the exotic and native ranges of *B. impatiens*.

| 2→1 | | | | |
| --- | --- | --- | --- | --- |
|  |  | NAw | Nat | CA |
| 2→1 | NAw | NA | 0.726 | 0 |
|  | Nat | 0.857 | NA | 0 |
|  | CA | 0.000 | 0.000 | NA |

Nat: Eastern North America native range; NAw: Western North America exotic range; CA: Central American exotic range.

**Table S5** – Pair‐wise niche expansion proportions between each of the exotic and native ranges of *B. impatiens*.

| 2→1 | | | | |
| --- | --- | --- | --- | --- |
|  |  | NAw | Nat | CA |
| 2→1  2→1 | NAw | NA | 0.274 | 1 |
|  | Nat | 0.143 | NA | 1 |
|  | CA | 1.000 | 1.000 | NA |

Nat: Eastern North America native range; NAw: Western North America exotic range; CA: Central American exotic range.

**Table S6** – Pair-wise niche overlap comparisons (D) between each of the exotic and native ranges of *B. ruderatus*.

|  | SA | Nat | Can | NZ |
| --- | --- | --- | --- | --- |
| SA | NA | NA | NA | NA |
| Nat | 0.057 | NA | NA | NA |
| Can | 0.001 | 0.002 | NA | NA |
| NZ | 0.123 | 0.125 | 0.046 | NA |

Nat: European native range; NZ: New Zealand exotic range; Can: Canary Islands exotic range; SA: South American exotic range.

**Table S7** – Pair‐wise niche similarity comparisons (p‐values) between each of the exotic and native ranges of *B. ruderatus*.

| 2→1 | | | | | |
| --- | --- | --- | --- | --- | --- |
|  |  | SA | Nat | Can | NZ |
| 2→1 | SA | NA | 0.257 | 0.495 | 0.149 |
|  | Nat | 0.267 | NA | 0.208 | 0.020 |
|  | Can | 0.317 | 0.208 | NA | 0.010 |
|  | NZ | 0.069 | 0.050 | 0.010 | NA |

Nat: European native range; NZ: New Zealand exotic range; Can: Canary Islands exotic range; SA: South American exotic range.

**Table S8** – Pair‐wise niche unfilling proportions between each of the exotic and native ranges of *B. ruderatus*.

| 2→1 | | | | | |
| --- | --- | --- | --- | --- | --- |
|  |  | SA | Nat | Can | NZ |
| 2→1 | SA | NA | 0.618 | 0.993 | 0.327 |
|  | Nat | 0.551 | NA | 0.987 | 0.236 |
|  | Can | 0.000 | 0.000 | NA | 0.000 |
|  | NZ | 0.071 | 0.196 | 0.980 | NA |

Nat: European native range; NZ: New Zealand exotic range; Can: Canary Islands exotic range; SA: South American exotic range.

**Table S9** – Pair‐wise niche stability proportions between each of the exotic and native ranges *B. ruderatus*.

| 2→1 | | | | | | |
| --- | --- | --- | --- | --- | --- | --- |
|  |  |  | SA | Nat | Can | NZ |
| 2→1 | | SA | NA | 0.449 | 1 | 0.929 |
|  |  | Nat | 0.382 | NA | 1 | 0.804 |
|  |  | Can | 0.007 | 0.013 | NA | 0.020 |
|  |  | NZ | 0.673 | 0.764 | 1 | NA |

Nat: European native range; NZ: New Zealand exotic range; Can: Canary Islands exotic range; SA: South American exotic range.

**Table S10** – Pair‐wise niche expansion proportions between each of the exotic and native ranges of *B. ruderatus*.

| 2→1 | | | | | | |
| --- | --- | --- | --- | --- | --- | --- |
|  |  |  | SA | Nat | Can | NZ |
| 2→1 | | SA | NA | 0.551 | 0 | 0.071 |
|  |  | Nat | 0.618 | NA | 0 | 0.196 |
|  |  | Can | 0.993 | 0.987 | NA | 0.980 |
|  |  | NZ | 0.327 | 0.236 | 0 | NA |

Nat: European native range; NZ: New Zealand exotic range; Can: Canary Islands exotic range; SA: South American exotic range.

**Table S11** – Pair-wise niche overlap comparisons (D) between each of the exotic and native ranges of *B. subterraneus*.

|  | NZ | Nat |
| --- | --- | --- |
| NZ | NA | NA |
| Nat | 0.149 | NA |

Nat: European native range; NZ: New Zealand exotic range.

**Table S12** – Pair‐wise niche similarity comparisons (p‐values) between each of the exotic and native ranges of *B. subterraneus*.

| 2→1 | | | | |
| --- | --- | --- | --- | --- |
|  |  |  | NZ | Nat |
|  |  | NZ | NA | 0.03 |
|  |  | Nat | 0.05 | NA |

Nat: European native range; NZ: New Zealand exotic range.

**Table S13** – Pair‐wise niche unfilling proportions between each of the exotic and native ranges of *B. subterraneus*.

| 2→1 | | | | |
| --- | --- | --- | --- | --- |
|  |  |  | NZ | Nat |
| 2→1 | | NZ | NA | 0.47 |
|  |  | Nat | 0.704 | NA |

Nat: European native range; NZ: New Zealand exotic range.

**Table S14** – Pair‐wise niche stability proportions between each of the exotic and native ranges of *B. subterraneus*.

| 2→1 | | | | |
| --- | --- | --- | --- | --- |
|  |  |  | NZ | Nat |
| 2→1 | | NZ | NA | 0.296 |
|  |  | Nat | 0.53 | NA |

Nat: European native range; NZ: New Zealand exotic range.

**Table S15** – Pair‐wise niche expansion proportions between each of the exotic and native ranges of *B. subterraneus*.

| 2→1 | | | | |
| --- | --- | --- | --- | --- |
|  |  |  | NZ | Nat |
| 2→1 | | NZ | NA | 0.704 |
|  |  | Nat | 0.47 | NA |

Nat: European native range; NZ: New Zealand exotic range.

**Table S16** – Pair-wise niche overlap comparisons (D) between each of the exotic and native ranges of *B. terrestris*.

|  | SA | NZ | Tas | Nat | ASe |
| --- | --- | --- | --- | --- | --- |
| SA | NA | NA | NA | NA | NA |
| NZ | 0.193 | NA | NA | NA | NA |
| Tas | 0.138 | 0.455 | NA | NA | NA |
| Nat | 0.119 | 0.259 | 0.398 | NA | NA |
| ASe | 0.004 | 0.011 | 0.000 | 0.101 | NA |

Nat: European native range; NZ: New Zealand exotic range; Tas: Tasmanian exotic range; SA: South American exotic range; ASe1: East Asian exotic range.

**Table S17** – Pair‐wise niche similarity comparisons (p‐values) between each of the exotic and native ranges of *B. terrestris*.

| 2→1 | | | | | | | |
| --- | --- | --- | --- | --- | --- | --- | --- |
|  |  |  | SA | NZ | Tas | Nat | ASe |
| 2→1 | | SA | NA | 0.158 | 0.03 | 0.069 | 0.772 |
|  |  | NZ | 0.079 | NA | 0.01 | 0.020 | 0.574 |
|  |  | Tas | 0.030 | 0.010 | NA | 0.010 | 1.000 |
|  |  | Nat | 0.040 | 0.020 | 0.01 | NA | 0.050 |
|  |  | ASe | 0.832 | 0.545 | 1.00 | 0.129 | NA |

Nat: European native range; NZ: New Zealand exotic range; Tas: Tasmanian exotic range; SA: South American exotic range; ASe1: East Asian exotic range.

**Table S18** – Pair‐wise niche unfilling proportions between each of the exotic and native ranges of *B. terrestris*.

| 2→1 | | | | | | | |
| --- | --- | --- | --- | --- | --- | --- | --- |
|  |  |  | SA | NZ | Tas | Nat | ASe |
| 2→1 | | SA | NA | 0.261 | 0.420 | 0.393 | 0.989 |
|  |  | NZ | 0.072 | NA | 0.086 | 0.355 | 0.996 |
|  |  | Tas | 0.025 | 0.000 | NA | 0.203 | 1.000 |
|  |  | Nat | 0.280 | 0.284 | 0.672 | NA | 0.652 |
|  |  | ASe | 0.918 | 0.912 | 1.000 | 0.142 | NA |

Nat: European native range; NZ: New Zealand exotic range; Tas: Tasmanian exotic range; SA: South American exotic range; ASe1: East Asian exotic range.

**Table S19** – Pair‐wise niche stability proportions between each of the exotic and native ranges of *B. terrestris*.

| 2→1 | | | | | | | |
| --- | --- | --- | --- | --- | --- | --- | --- |
|  |  |  | SA | NZ | Tas | Nat | ASe |
| 2→1 | | SA | NA | 0.928 | 0.975 | 0.720 | 0.082 |
|  |  | NZ | 0.739 | NA | 1.000 | 0.716 | 0.088 |
|  |  | Tas | 0.580 | 0.914 | NA | 0.328 | 0.000 |
|  |  | Nat | 0.607 | 0.645 | 0.797 | NA | 0.858 |
|  |  | ASe | 0.011 | 0.004 | 0.000 | 0.348 | NA |

Nat: European native range; NZ: New Zealand exotic range; Tas: Tasmanian exotic range; SA: South American exotic range; ASe1: East Asian exotic range.

**Table S20** – Pair‐wise niche expansion proportions between each of the exotic and native ranges of *B. terrestris*.

| 2→1 | | | | | | | |
| --- | --- | --- | --- | --- | --- | --- | --- |
|  |  |  | SA | NZ | Tas | Nat | ASe |
| 2→1 | | SA | NA | 0.072 | 0.025 | 0.280 | 0.918 |
|  |  | NZ | 0.261 | NA | 0.000 | 0.284 | 0.912 |
|  |  | Tas | 0.420 | 0.086 | NA | 0.672 | 1.000 |
|  |  | Nat | 0.393 | 0.355 | 0.203 | NA | 0.142 |
|  |  | ASe | 0.989 | 0.996 | 1.000 | 0.652 | NA |

Nat: European native range; NZ: New Zealand exotic range; Tas: Tasmanian exotic range; SA: South American exotic range; ASe1: East Asian exotic range.

**Table S21.** Bioclimatic variables with higher correlation in the potential distribution model.

| **BIOCLIMATIC VARIABLES** | **CP1** | **CP2** | **CP3** |
| --- | --- | --- | --- |
| Bio1: Annual Mean Temperature | **0.2784** | 0.2150 | 0.0447 |
| Bio10: Mean Temperature of Warmest Quarter | 0.2557 | **0.2609** | 0.1636 |
| Bio11: Mean Temperature of Coldest Quarter | **0.2876** | 0.1644 | -0.0680 |
| Bio12: Annual Precipitation | 0.2512 | **-0.2803** | 0.0589 |
| Bio14: Precipitation of Driest Month | 0.1744 | **-0.3176** | **0.2796** |
| Bio15: Precipitation Seasonality (Coefficient of Variation) | -0.1200 | 0.0221 | **-0.4387** |
| Bio17: Precipitation of Driest Quarter | 0.1824 | **-0.3248** | **0.2658** |
| Bio19: Precipitation of Coldest Quarter | 0.1796 | **-0.3056** | 0.0468 |
| Bio2: Mean Diurnal Range (Mean of monthly (↑T° - ↓T°)) | 0.1207 | **0.3699** | -0.0164 |
| Bio4: Temperature Seasonality (standard deviation ×100) | -0.2147 | 0.0831 | **0.4441** |
| Bio5: Max Temperature of Warmest Month | 0.2493 | **0.2799** | 0.1686 |
| Bio6: Min Temperature of Coldest Month | **0.2892** | 0.1442 | -0.0693 |
| Bio7: Temperature Annual Range (BIO5-BIO6) | -0.1772 | 0.1884 | **0.4486** |
| Bio9: Mean Temperature of Driest Quarter | **0.2704** | 0.1619 | -0.1367 |

Bold values indicate correlations with more significant effects (r > ± 0.25).

## **Supplementary Figures**

**Figure S1.** PCA‐env results and the relationship of each environmental variable related to the distribution of *B. impatiens* in all its native and introduced ranges. **A)** The first two PCA axes and the orientation of each environmental variable in each axis. **B)** Individual contribution of each environmental variable to the first PCA axis. **C)** Individual contribution of each environmental variable to the second PCA axis.


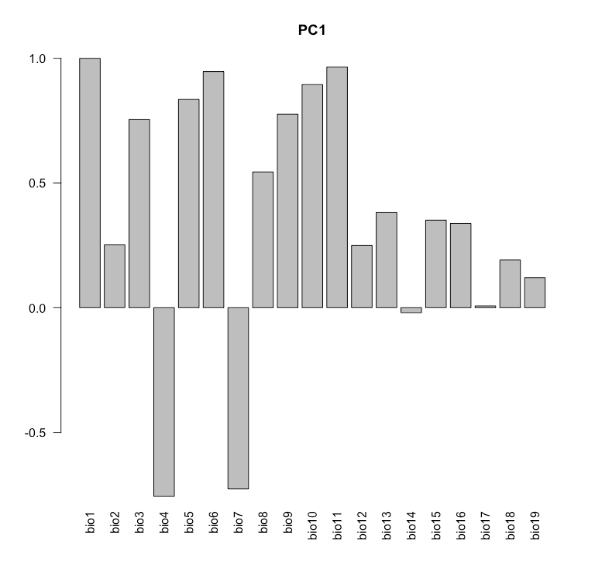

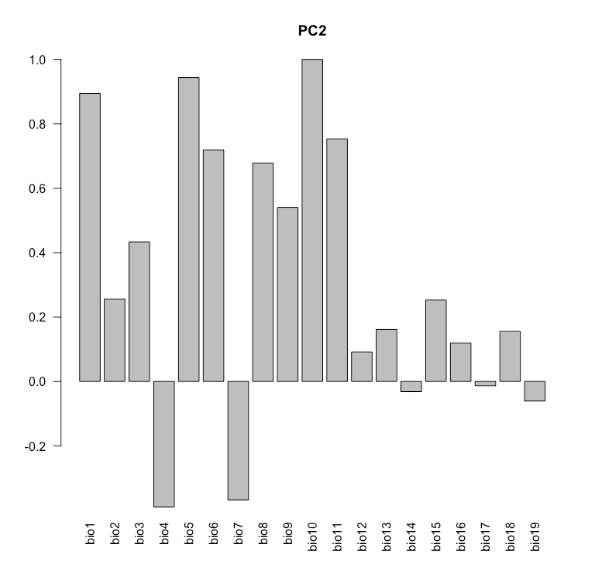

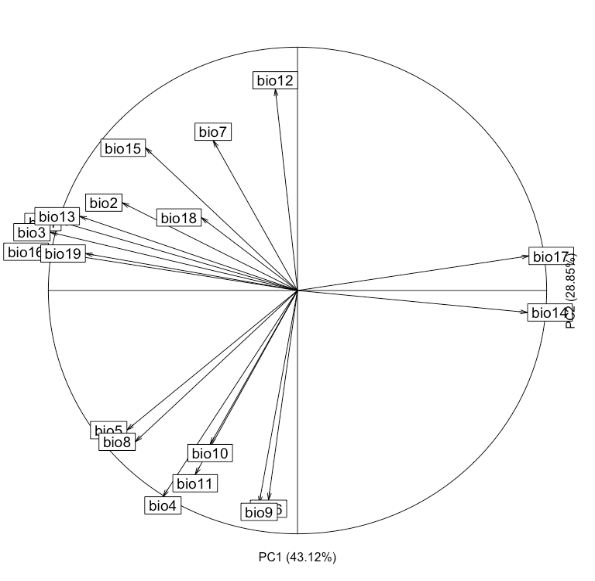


**A)**

**B)**

**C)**

**Figure S2.** PCA‐env results and the relationship of each environmental variable related to the distribution of *B. ruderatus* in all its native and introduced ranges. **A)** The first two PCA axes and the orientation of each environmental variable in each axis. **B)** Individual contribution of each environmental variable to the first PCA axis. **C)** Individual contribution of each environmental variable to the second PCA axis.


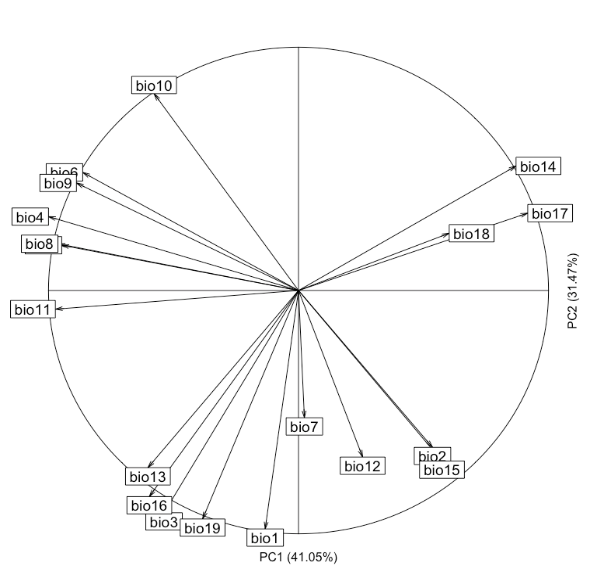

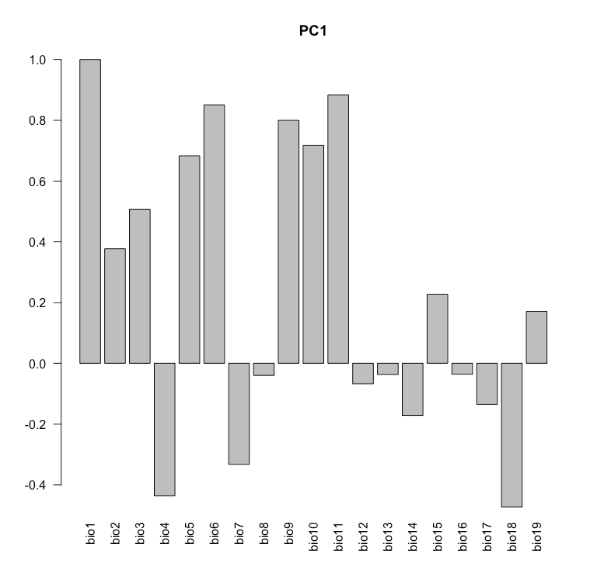

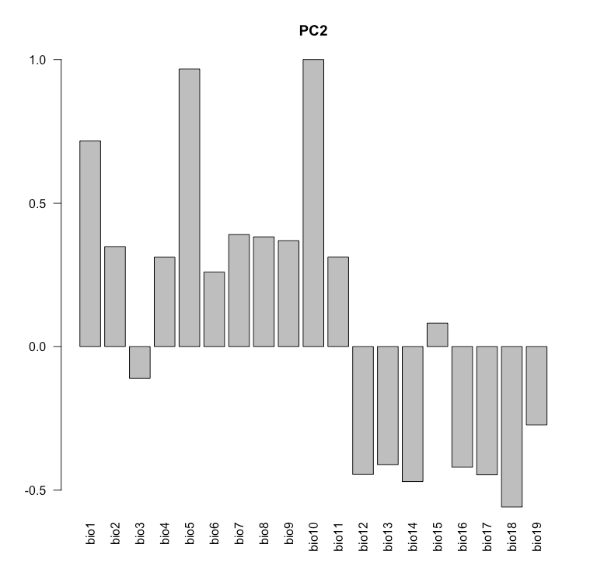


**C)**

**A)**

**B)**

**Figure S3.** PCA‐env results and the relationship of each environmental variable related to the distribution of *B. subterraneus* in all its native and introduced ranges. **A)** The first two PCA axes and the orientation of each environmental variable in each axis. **B)** Individual contribution of each environmental variable to the first PCA axis. **C)** Individual contribution of each environmental variable to the second PCA axis.


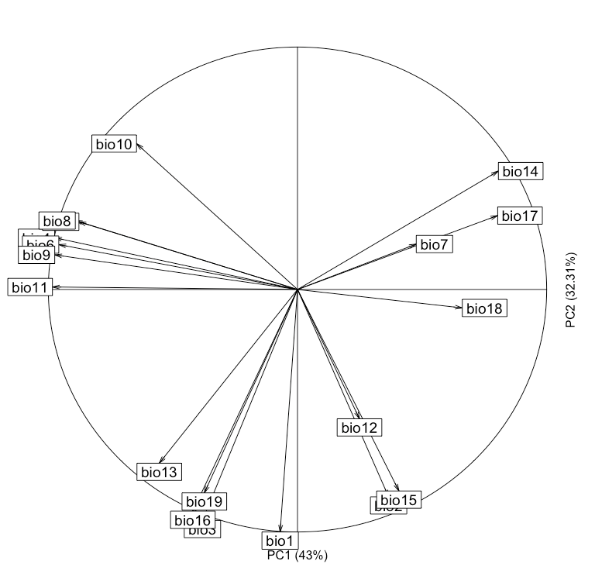

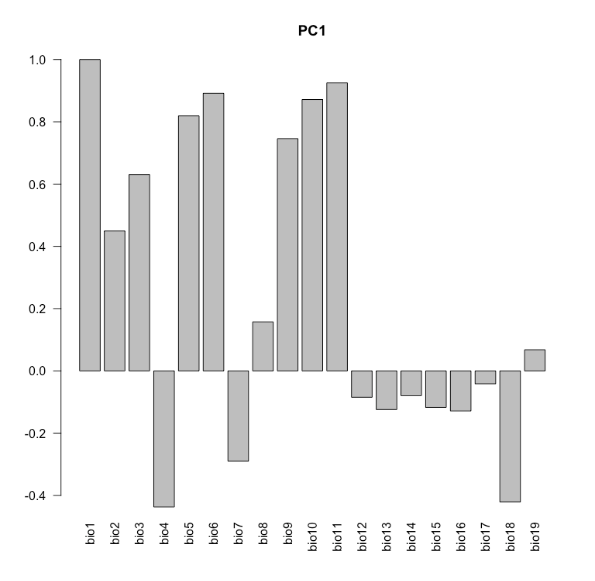

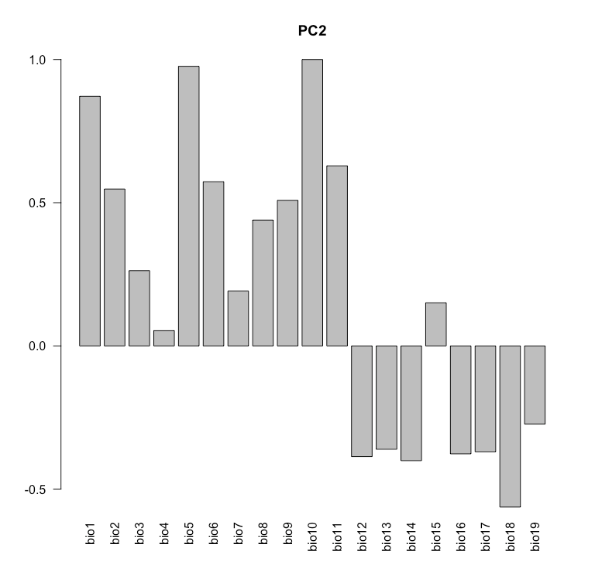


**A)**

**B)**

**C)**

**Figure S4.** PCA‐env results and the relationship of each environmental variable related to the distribution of *B. terrestris* in all its native and introduced ranges. **A)** The first two PCA axes and the orientation of each environmental variable in each axis. **B)** Individual contribution of each environmental variable to the first PCA axis. **C)** Individual contribution of each environmental variable to the second PCA axis.


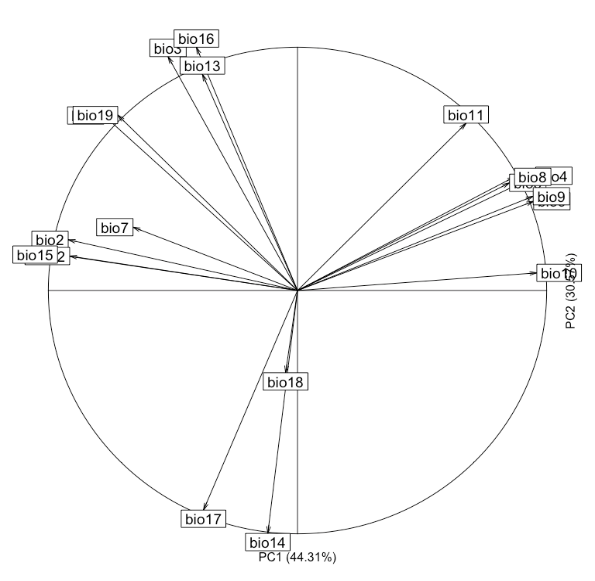

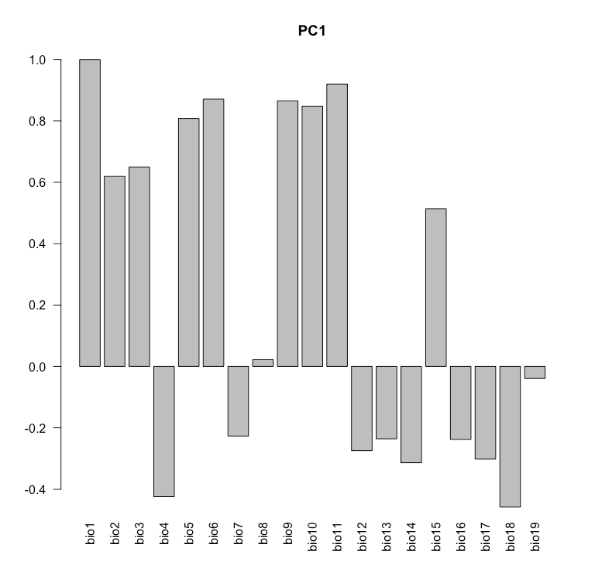

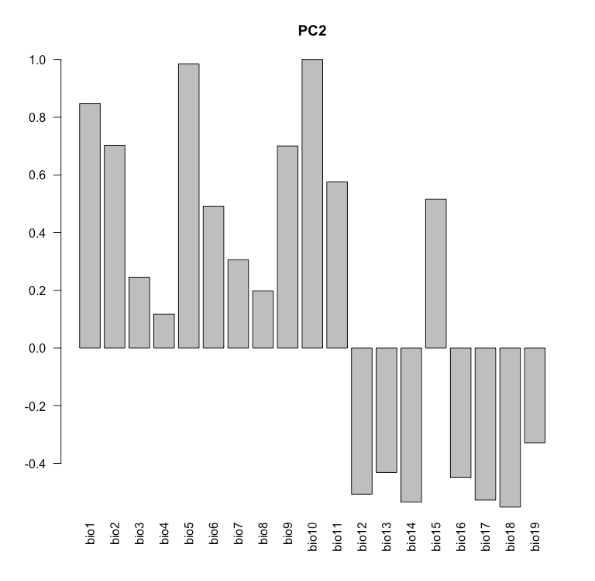


**A)**

**B)**

**C)**


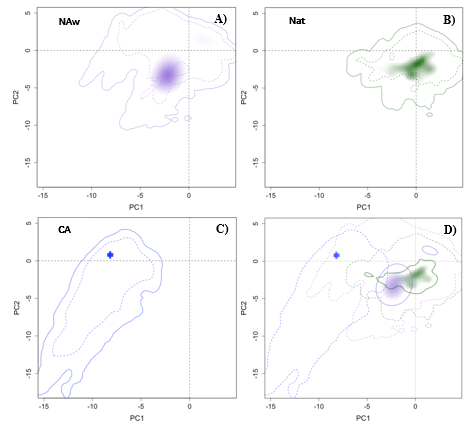


**Figure S5.** Climatic conditions occupied by *B. impatiens*, A) Western North America, B) Native Area, C) Central America, and D) overlap of all areas. Continuous and discontinuous lines represent 100% and 50%, respectively, of available climate.


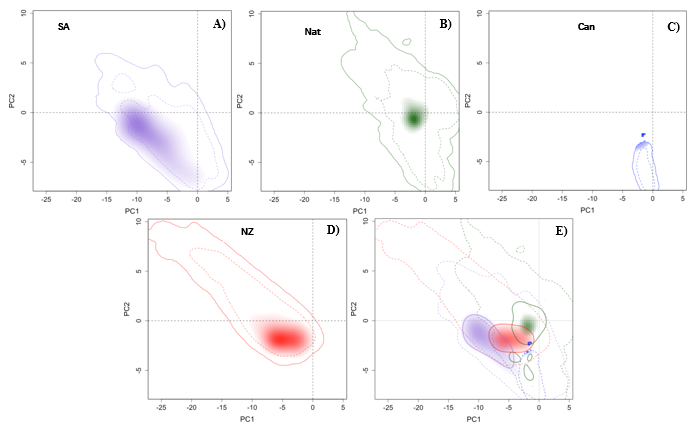


**Figure S6.** Climatic conditions occupied by *B. ruderatus*, A) South America, B) Native Area, C) Canary Islands, D) New Zealand, E) overlap of all areas. Continuous and discontinuous lines represent 100% and 50%, respectively, of available climate.


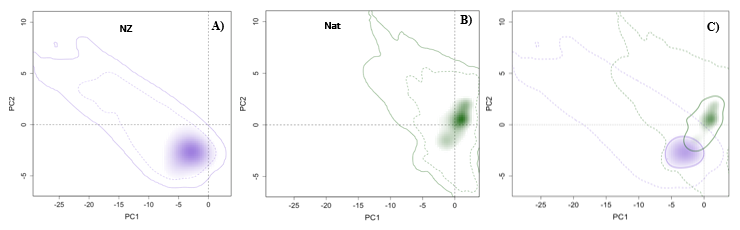


**Figure S7.** Climatic conditions occupied by *B. subterraneus,* A) New Zealand, B) Native Area, C) overlap of all areas. Continuous lines and discontinuous lines represent 100% and 50%, respectively, of available climate.


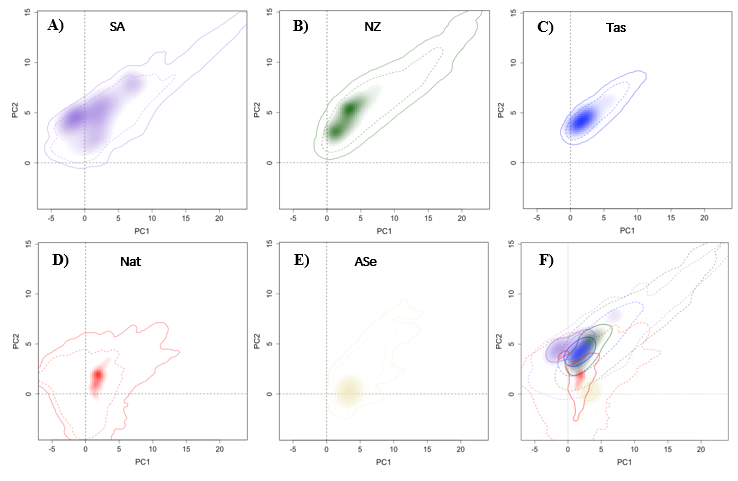


**Figure S8.** Climate conditions occupied by B. terrestris, A) South America, B) New Zealand, C) Tasmania, D) Native Area, E) East Asia, and F) overlapping of all areas. Continuous lines and discontinuous lines represent 100% and 50%, respectively, of available climate.

## **Code description *Bombus* spp.**

**Before starting**

To perform the models and analyses, we recommend all users check the latest version of R at <http://www.r-project.org/> and ensure they are using the updated versions of their installed R packages. Users can automatically update their installed packages with the following code:

### Species Distribution Models (SDM)

These species distribution models are framed in the ENMTML package developed by Andrade et al. (2020)*,* and the methods and algorithms used were defined according to the model requirements for these species (see methods section in the manuscript).

# *Download and install from Google the RTools 64-bits*

*#* (<https://cran.r-project.org/bin/windows/Rtools/rtools40.html>)

*#* *get work directory*

setwd("C:/Models")

getwd()

The ENMTML package has dependencies on different R packages available in CRAN. Use the following code to install them.

*#* *install.packages("units", repos= "*https://cran.r-project.org/*")*

*#* *install.packages("udunits", repos= "*https://cran.r-project.org/*")*

*# install.packages("udunits2")*

*# if (!"devtools"%in%installed.packages()){install.packages("devtools")}*

*#* devtools::install_github("andrefaa/ENMTML",force=T)

Once installed, load them.

# Load packages

library(units)

library(udunits2)

library(ENMTML)

require(ENMTML)

The help function of the package shows the arguments to be specified according to the modeling requirements.

?ENMTML

The folder directories are edited according to each species. Here, loading the bioclimatic variables and the occurrence points is necessary.

ENMTML(

pred_dir = "C:/Models/Variables/Current",

proj_dir = NULL,

result_dir = "C:/Models/Results/species",

occ_file = "C:/Models/Occurrences/species.txt",

sp = "ScientificName",

x = "Long",

y = "Lat",

min_occ = 10,

thin_occ = c(method='CELLSIZE'),

eval_occ = NULL,

colin_var = c(method='PCA'),

imp_var = FALSE,

sp_accessible_area = NULL,

pseudoabs_method = c(method= 'ENV_CONST'),

pres_abs_ratio = 1,

part = c(method= 'BLOCK'),

save_part = FALSE,

save_final = TRUE,

algorithm = c(“MXS”, “SVM”, “BRT”, “RDF”),

thr = c(type='JACCARD',type='LPT'),

msdm = c(method='OBR'),

ensemble = NULL,

extrapolation = FALSE,

cores = 2

### Niche Analysis

# Update installed packages
# update.packages(checkBuilt = TRUE, ask = FALSE)

The analysis presented here uses the following R packages available at CRAN. Use the following code to install them.

# install packages
# install.packages("knitr")
# install.packages("spThin")
# install.packages("rgeos")
# install.packages("sp")
# install.packages("maptools")
# install.packages("raster")
# install.packages("ecospat")
# install.packages("here")
# install.packages("tidyverse")
# install.packages("rnaturalearth")
# install.packages("stars")
# install.packages("sf")

Once installed, load them.

# Load packages
library(knitr)
library(spThin)
library(rgeos)
library(sp)
library(maptools)
library(raster)
library(ecospat)
library(here)
library(tidyverse)
library(sf)
library(rnaturalearth)
library(stars)
library(readxl)
library(colorspace)

##

## **Data**

**Load the occurrence records**

Load the occurrence records into the R environment (edit a table with the records of each species when you run the code).

occ.points <- read.csv(here("data", "B. impatiens.csv"))
colnames(occ.points)[2:5] <- c(“Latitude”, “Longitude”, “Distribution”, “Species”)

The loaded table for: *B. impatiens* includes 122,257 occurrence records, *B.ruderatus* 2,950, *B. subterraneus* 5,173 and *B. terrestris* 76,233.

**Thinning occurrence records**

Leave only one point in each cell.

r <- raster(res = 0.1)
values(r) <- rep(1, ncell(r))
species <- unique(occ.points$Species)
keep <- numeric(0)
for (i in 1:length(species)) {
 where <- occ.points$Species == species[i]
 occ.points_temp <- occ.points[where, ]
 if (nrow(occ.points_temp) > 1000) {
 locs <- raster::extract(r, occ.points_temp[, 3:2], cellnumbers = TRUE)[, 1]
 keep <- c(keep, which(!duplicated(locs)))
 } else {
 keep <- c(keep, which(where))
 }
}
occ.points2 <- occ.points[keep, ]

The occurrence records gathered (see the methods section of the manuscript for the description of how we obtained the data) are not free from geographical sample bias. To minimize this problem, we applied a thinning procedure using the **spThin** package to ensure that all the points have a minimum distance of 10km from each other (see Aiello-Lammens et al. 2014 for the algorithm description).

occ.points.thin <- list()
for (i in 1:length(species)) {
 occ.points.thin[[i]] <- occ.points2 %>%
 filter(Species == species[i]) %>%
 thin(
 verbose = FALSE,
 lat.col = "Latitude",
 long.col = "Longitude",
 spec.col = "Species",
 thin.par = 5,
 reps = 1,
 write.files = FALSE,
 write.log.file = FALSE,
 locs.thinned.list.return = TRUE
 )
}
occ.points.thin <- lapply(occ.points.thin, "[[", 1)
names(occ.points.thin) <- species

After the thinning procedure, the number of occurrence points is reduced for each species to *B. impatiens* = NAw 73, Nat 4449, CA 13, *B. ruderatus*= SA 76, Nat 816, Can 10, NZ 130, *B. subterraneus*= NZ 28, Nat 995, and *B. terrestris*= SA 241, NZ 461, Tas 164, Nat 5216, ASe 113.

**Define the regions to be tested**

The first step is to define the number of groups (regions) to be tested. In the following case, we chose three groups for *B. impatiens,* 4 for *B. ruderatus*, 2 for *B. subterraneus,* and 5 for *B. terrestris.*

n.groups <- length(species)

Now, define the name of the groups in the same geographical order of the groups, starting from west to east. You can also define the codes to be used in the tables.

g.names <- species
g.codenames <- g.names

It is also necessary to set what colors will be used in the following plots for each group (using the same order as the names). Change the colors according to your preferences.

g.colors <- c(“mediumpurple”, ‘darkgreen’, ‘blue’, ‘red’,
 “lemonchiffon2”, “deeppink”, “black”,
 “darkorchid4”, “gold”, “brown”, “orange”)

To check the distribution of the occurrence records, we map them in a world context.

occ.points.thin2 <- do.call(rbind, occ.points.thin) %>%
 tibble::rownames_to_column(var = "species") %>%
 mutate(species = gsub("\\..*", "", species),
 species = factor(species, g.names))
world <- ne_countries(scale = "medium", returnclass = "sf")
g <- ggplot() +
 geom_sf(data = world) +
 theme_minimal() +
 geom_point(occ.points.thin2, mapping = aes(Longitude, Latitude,
 col = species),
 size = .3) +
 scale_color_manual(values = g.colors) +
 xlab("") +
 ylab("") +
 theme(
 strip.text = element_text(face = "italic"),
 legend.text = element_text(face = "italic"),
 legend.position = "none"
 )
g1 <- g + facet_wrap(species ~ ., ncol = 3, nrow = 4)
g1

*B. impatiens*


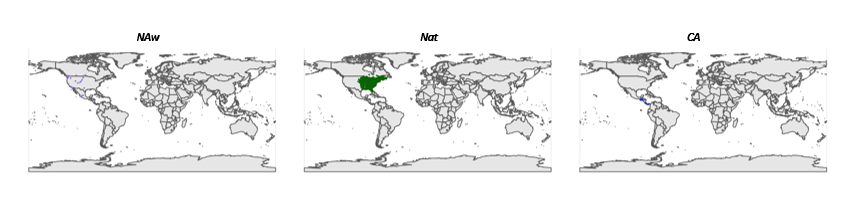


*B. ruderatus*


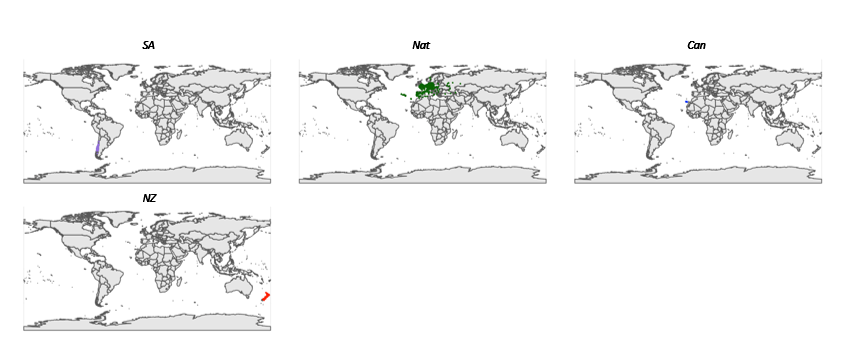


*B. subterraneus*


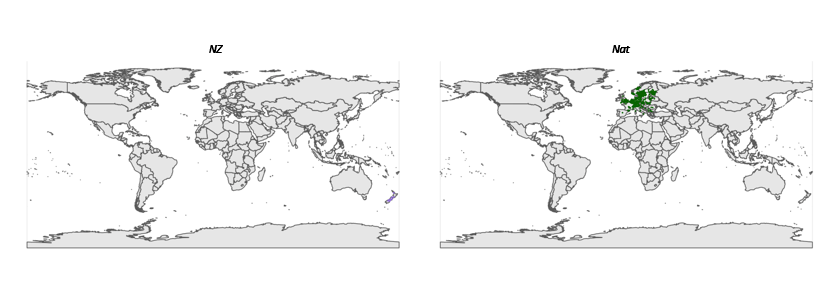


*B. terrestris*


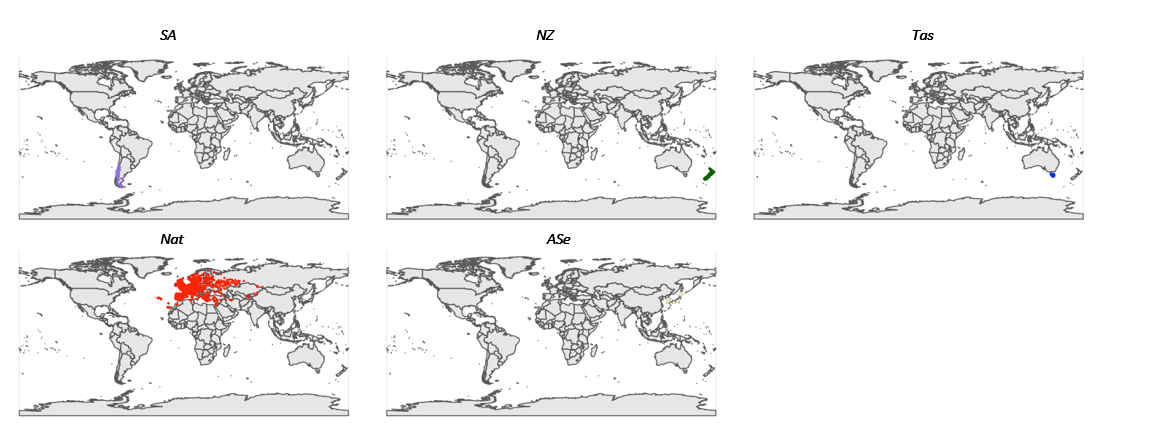


loc <- here("Figures", "Figure1.tiff")
# ggsave(loc, g1)

Filter points to the countries defined.

pnts_sf <- st_as_sf(occ.points.thin2,
 coords = c(‘Longitude’, ‘Latitude’),
 crs = st_crs(map))
# pnts <- pnts_sf %>%
# mutate(intersection = as.integer(st_intersects(geometry, map)))
# remove <- is.na(pnts$intersection)
occ.points.thin3 <- occ.points.thin2
g <- ggplot() +
 geom_sf(data = world) +
 theme_minimal() +
 geom_point(occ.points.thin3, mapping = aes(Longitude, Latitude,
 col = species),
 size = .3) +
 scale_color_manual(values = g.colors) +
 xlab("") +
 ylab("") +
 theme(
 strip.text = element_text(face = "italic"),
 legend.text = element_text(face = "italic"),
 legend.position = "none",
 axis.text.x = element_text(angle = 45, hjust = 1))

g1 <- g + facet_wrap(species ~ ., ncol = 4, nrow = 3)
g1

*B. impatiens*

####
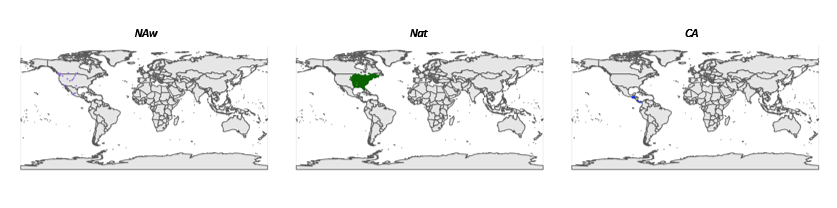


*B. ruderatus*


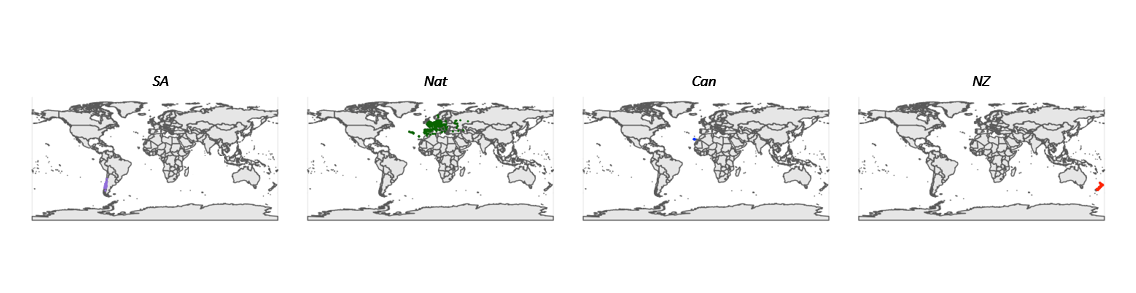


*B. subterraneus*


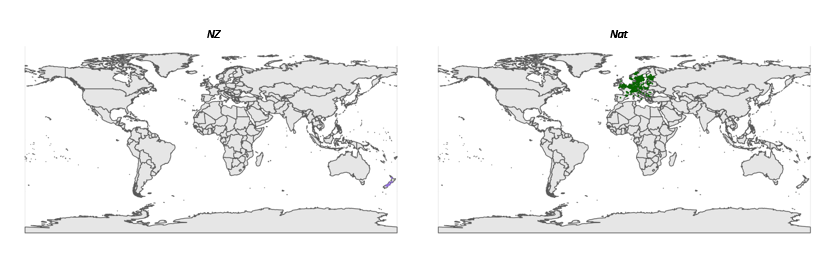


*B. terrestris*


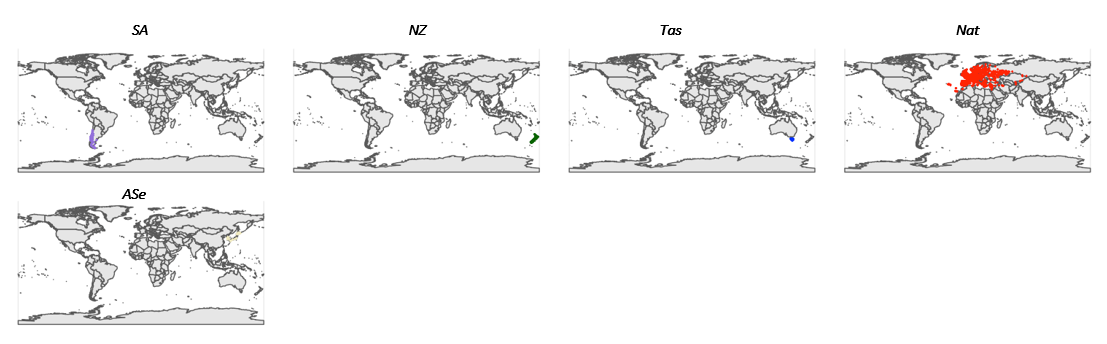


loc <- here("Figures", "Figure2.tiff")
# ggsave(loc, g1)

**Background definition**

An essential step in the niche analysis is the definition of the background. Here, we applied a background based on a minimum convex polygon (MCP) made from the occurrence records of each group. Additionally to the MCP, we add a buffer around it. The polygon buffer size for the background (in degrees) can be changed below. We chose 2 degrees based on the species dispersion.

buffer.size <- 2

We define a minimum convex polygon (MCP) function below (this function was obtained from <https://github.com/ndimhypervol/wallace>).

mcp <- function (xy) {
 xy <- as.data.frame(coordinates(xy))
 coords.t <- chull(xy[, 1], xy[, 2])
 xy.bord <- xy[coords.t, ]
 xy.bord <- rbind(xy.bord[nrow(xy.bord), ], xy.bord)
 return(SpatialPolygons(list(Polygons(list(Polygon(as.matrix(xy.bord))), 1))))
}

**Environmental variables**

The environmental variables used are available on the WorldClim website (<http://www.worldclim.org>). Download all the 19 bioclimatic (‘Biolclim’) variables for the current conditions (we used the resolution of 10 arc-min) with the code below. Note you need to have the internet on. The download files are opened and directed in the R environment, but they are also saved in your work directory (to see where it is, use getwd()).

variables <- raster::stack(list.files(
 here("pres"),
 full.names = TRUE))

In the manuscript, we used all the 19 bioclimatic variables as before the analysis. We will reduce them to a two-dimensional space with a PCA. However, the readers can choose the number of variables to keep by changing the sequence 1:19 in the code below for the variable number you want to keep (to see the name sequence of the variables, apply names(variables)).

variables <- subset(variables, 1:nlayers(variables))

You can also check the variables by mapping them.

plot(variables)


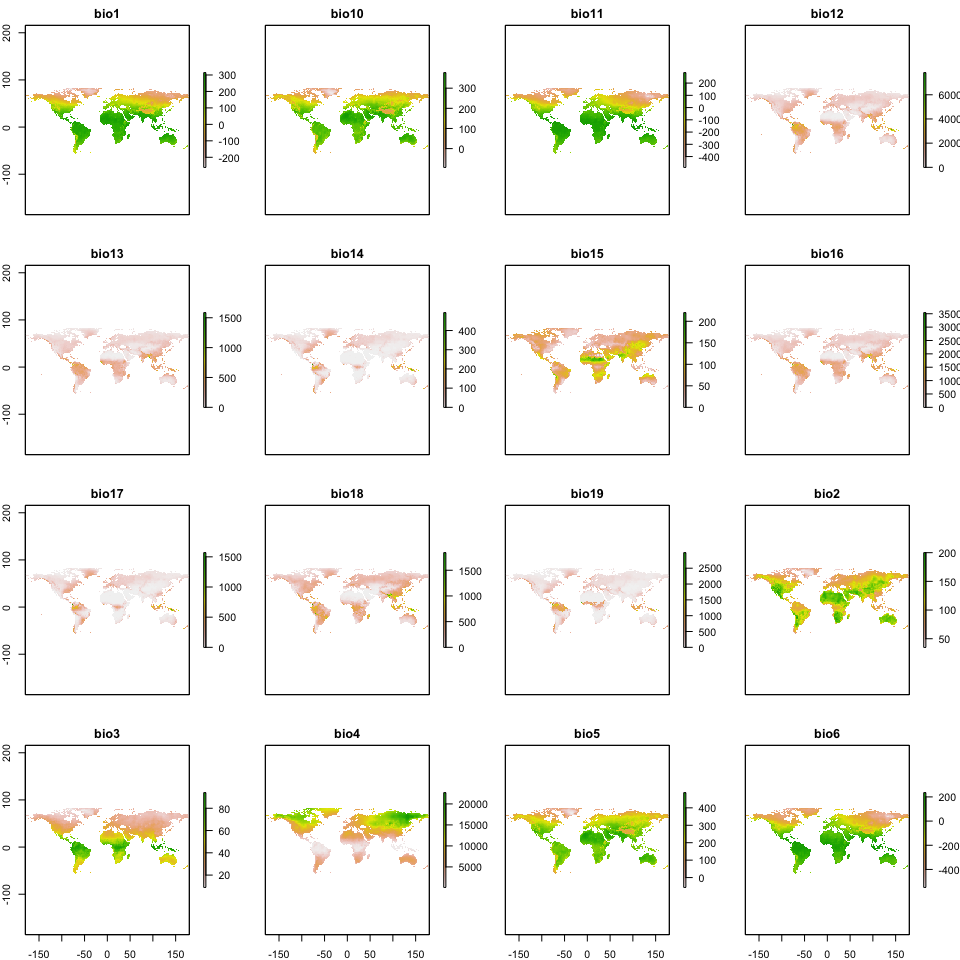


**Group assigning**

Once we have the occurrence data, the environmental data, the defined groups, and their background parameters chosen, we can prepare the data for the analysis. Below, we use the occurrence points to generate the MCP plus a buffer defined by the user for the background (see above). Next, the variable values per group are extracted from the species occurrence points and the background (defined above). Finally, we plot the resulting groups with their respective backgrounds.

# Empty objects
g.assign <- occ.points.thin3$species
xy.mcp <- list()
back.env <- list()
spec.env <- list()
row.sp <- list()
united <- st_union(st_make_valid(world))
# Loop
for (i in 1:n.groups) {
 print(i)
 # Save row numbers per species
 g.limit <- g.assign == species[i]
 row.sp[[i]] <- which(g.limit)
 # Background polygon
 mcp.occ <- mcp(as.matrix(occ.points.thin3[g.limit, -1]))
 xy.mcp.i <- gBuffer(mcp.occ, width = buffer.size) %>%
 st_as_sf() %>%
 st_set_crs(value = st_crs(world))

xy.mcp[[i]] <- st_intersection(xy.mcp.i, united)
 xy.mcp[[i]]$species <- species[i]
 # Background environment
 extract_temp <- raster::extract(variables, as_Spatial(xy.mcp[[i]]))
 back.env[[i]] <- na.exclude(do.call(rbind.data.frame,
 extract_temp))
 # Species environment
 spec.env[[i]] <- na.exclude(raster::extract(variables,
 occ.points.thin3[g.limit, -1]))
}

## [1] 1
## [1] 2
## [1] 3

Map buffers:

xy.mcp.is <- do.call(rbind, xy.mcp)
xy.mcp.is$species <- factor(xy.mcp.is$species, xy.mcp.is$species)
g <- ggplot() +
 geom_sf(data = world) +
 theme_minimal() +
 geom_sf(data = xy.mcp.is, aes(col = species)) +
 geom_point(occ.points.thin3, mapping = aes(Longitude, Latitude,
 col = species),
 size = .3) +
 scale_color_manual(values = g.colors) +
 xlab("") +
 ylab("") +
 theme(
 strip.text = element_text(face = "italic"),
 legend.text = element_text(face = "italic"),
 legend.position = "none",
 axis.text.x = element_text(angle = 45, hjust = 1))
g1 <- g + facet_wrap(species ~ ., ncol = 3, nrow = 4)
g1

*B. impatiens*


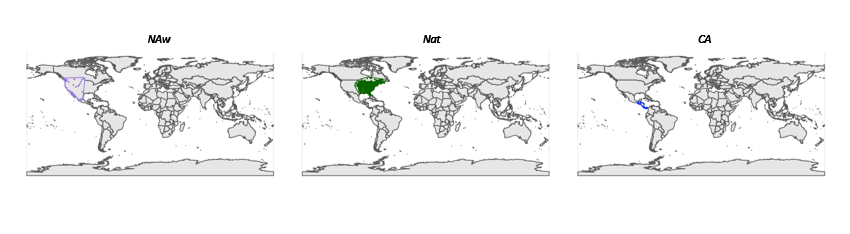


*B. ruderatus*


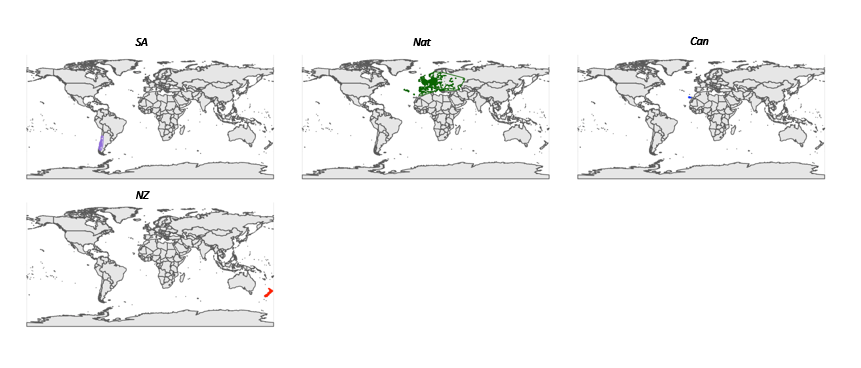


*B. subterraneus*


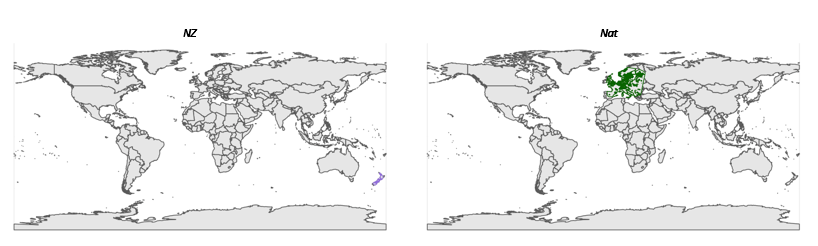


*B. terrestris*


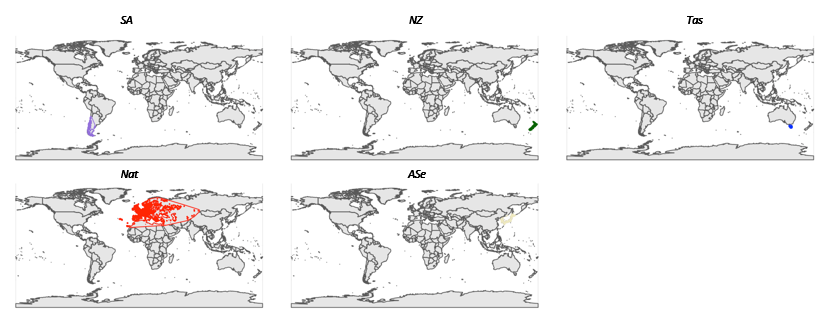


loc <- here(“Figures”, “Figure.tiff”)
#ggsave(loc, g1)

Save the occurrence points table.

write.csv(occ.points.thin3,
 file = here(“Data”, “occurrences.csv”),
 row.names = FALSE)

Now, we organize the final tables to be used.

# Occurrence points per group
g.occ.points <- occ.points.thin3
colnames(g.occ.points)[1] <- "Groups"
# Environmental values for the background
all.back.env <- do.call(rbind.data.frame, back.env)
# Environmental values for the species occurrence points
all.spec.env <- do.call(rbind.data.frame, spec.env)
# Environmental values all together
data.env <- rbind(all.spec.env, all.back.env)

Check the number of occurrence records by region for species.

table(g.occ.points[, 1]) B. impatiens

NAw Nat CA

73 4449 13

table(g.occ.points[, 1]) B. ruderatus

SA Nat Can NZ
76 816 10 130

table(g.occ.points[, 1]) B. subterraneus

NZ Nat

28 995

table(g.occ.points[, 1]) B. terrestris

SA NZ Tas Nat ASe
241 461 164 5216 113

**Niche comparisons**

The niche analyses and comparisons follow the framework developed by Broennimann et al. (2012) and its derivations (see methods section in the manuscript).

**PCA**

We applied a PCA (Principal Component Analysis), considering all the environments together, as it presented the best performance when comparing the niches (Broennimann et al., 2012).

# Weight matrix
w <- c(rep(0, nrow(all.spec.env)), rep(1, nrow(all.back.env)))
# PCA of all environment
pca.cal <- dudi.pca(data.env, row.w = w, center = TRUE,
 scale = TRUE, scannf = FALSE, nf = 2)

Once we have the PCA results, we need the first and second eigenvector values for the background and the occurrence records per group.

# Rows in data corresponding to sp1
adtion <- cumsum(c(0, sapply(back.env, nrow)))
begnd <- nrow(all.spec.env)
# Empty list to save the results
scores.back <- list()
scores.spec <- list()
# Assigning the values
for (i in 1:n.groups) {
 scores.spec[[i]] <- pca.cal$li[row.sp[[i]], ]
 pos <- (begnd[1] + adtion[i] + 1) : (begnd[1] + adtion[i + 1])
 scores.back[[i]] <- pca.cal$li[pos, ]
}
total.scores.back <- do.call(rbind.data.frame, scores.back)

**Environmental space**

An environmental space is generated based on the PCA values calculated for the background and the occurrence records. We defined the resolution of this two-dimensional space grid below.

R <- 100

Next, we modeled the species density in the environmental grid, considering the observed occurrence density and the availability of the conditions in the background.

z <- list()
for (i in 1:n.groups) {
 z[[i]] <- ecospat.grid.clim.dyn(total.scores.back,
 scores.back[[i]],
 scores.spec[[i]],
 R = R)
}

**Niche overlap**

We calculate the D metric and its significance for the niche overlap using a similarity test. We define the number of interactions for the similarity test below (see the methods section in the manuscript for details).

rep <- 100

Once the number of interactions is defined, we can generate the values. Additionally, we calculate the partition of the non-overlapped niche among niche unfilling, expansion, and stability (see methods in the manuscript).

# Empty matrices
D <- matrix(nrow = n.groups, ncol = n.groups)
rownames(D) <- colnames(D) <- substr(g.codenames, 1, 3)
unfilling <- stability <- expansion <- sim <- D
sim_unfilling <- sim_stability <- sim_expansion <- D
for (i in 2:n.groups) {
 for (j in 1:(i - 1)) {
 x1 <- z[[i]]
 x2 <- z[[j]]
 # Niche overlap
 D[i, j] <- ecospat.niche.overlap (x1, x2, cor = TRUE)$D
 # Niche similarity
 ps <- ecospat.niche.similarity.test(x1, x2, rep)
 ps2 <- ecospat.niche.similarity.test(x2, x1, rep)
 sim[i, j] <- ps$p.D
 sim[j, i] <- ps2$p.D
 sim_unfilling[i, j] <- ps$p.unfilling
 sim_unfilling[j, i] <- ps2$p.unfilling
 sim_stability[i, j] <- ps$p.stability
 sim_stability[j, i] <- ps2$p.stability
 sim_expansion[i, j] <- ps$p.expansion
 sim_expansion[j, i] <- ps2$p.expansion
 # Niche Expansion, Stability, and Unfilling
 index1 <- ecospat.niche.dyn.index (x1, x2,
 intersection = NA)$dynamic.index.w
 index2 <- ecospat.niche.dyn.index (x2, x1,
 intersection = NA)$dynamic.index.w
 expansion[i, j] <- index1[1]
 stability[i, j] <- index1[2]
 unfilling[i, j] <- index1[3]
 expansion[j, i] <- index2[1]
 stability[j, i] <- index2[2]
 unfilling[j, i] <- index2[3]
 }
}

## **Numeric results**

Below, we present the results for each metric among all the groups.

*B. impatiens*

Niche unfilling null model (p-values):

kable(sim_unfilling, digits = 3, format = "markdown")

|  | NAw | Nat | CA |
| --- | --- | --- | --- |
| NAw | NA | 0.059 | 1 |
| Nat | 0.139 | NA | 1 |
| CA | 1.000 | 1.000 | NA |

Niche expansion null model (p-values):

kable(sim_expansion, digits = 3, format = "markdown")

|  | NAw | Nat | CA |
| --- | --- | --- | --- |
| NAw | NA | 0.119 | 1 |
| Nat | 0.079 | NA | 1 |
| CA | 1.000 | 1.000 | NA |

Niche Stability null model (p-values):

kable(sim_stability, digits = 3, format = "markdown")

|  | NAw | Nat | CA |
| --- | --- | --- | --- |
| NAw | NA | 0.119 | 1 |
| Nat | 0.079 | NA | 1 |
| CA | 1.000 | 1.000 | NA |

*B. ruderatus*

Niche unfilling null model (p-values):

kable(sim_unfilling, digits = 3, format = "markdown")

|  | SA | Nat | Can | NZ |
| --- | --- | --- | --- | --- |
| SA | NA | 0.554 | 0.426 | 0.119 |
| Nat | 0.297 | NA | 0.079 | 0.040 |
| Can | 0.257 | 0.109 | NA | 0.010 |
| NZ | 0.079 | 0.099 | 0.010 | NA |

Niche expansion null model (p-values):

kable(sim_expansion, digits = 3, format = "markdown")

|  | SA | Nat | Can | NZ |
| --- | --- | --- | --- | --- |
| SA | NA | 0.307 | 0.356 | 0.109 |
| Nat | 0.545 | NA | 0.119 | 0.099 |
| Can | 0.257 | 0.040 | NA | 0.010 |
| NZ | 0.069 | 0.069 | 0.010 | NA |

Niche Stability null model (p-values):

kable(sim_stability, digits = 3, format = "markdown")

|  | SA | Nat | Can | NZ |
| --- | --- | --- | --- | --- |
| SA | NA | 0.307 | 0.356 | 0.109 |
| Nat | 0.545 | NA | 0.119 | 0.099 |
| Can | 0.257 | 0.040 | NA | 0.010 |
| NZ | 0.069 | 0.069 | 0.010 | NA |

*B. subterraneus*

Niche unfilling null model (p-values):

kable(sim_unfilling, digits = 3, format = "markdown")

|  | NZ | Nat |
| --- | --- | --- |
| NZ | NA | 0.178 |
| Nat | 0.277 | NA |

Niche expansion null model (p-values):

kable(sim_expansion, digits = 3, format = "markdown")

|  | NZ | Nat |
| --- | --- | --- |
| NZ | NA | 0.178 |
| Nat | 0.238 | NA |

Niche Stability null model (p-values):

kable(sim_stability, digits = 3, format = "markdown")

|  | NZ | Nat |
| --- | --- | --- |
| NZ | NA | 0.178 |
| Nat | 0.238 | NA |

*B. terrestris*

Niche unfilling null model (p-values):

kable(sim_unfilling, digits = 3, format = "markdown")

|  | SA | NZ | Tas | Nat | ASe |
| --- | --- | --- | --- | --- | --- |
| SA | NA | 0.257 | 0.03 | 0.267 | 0.861 |
| NZ | 0.178 | NA | 0.01 | 0.089 | 0.693 |
| Tas | 0.109 | 0.178 | NA | 0.030 | 1.000 |
| Nat | 0.149 | 0.050 | 0.02 | NA | 0.099 |
| ASe | 0.842 | 0.752 | 1.00 | 0.168 | NA |

Niche expansion null model (p-values):

kable(sim_expansion, digits = 3, format = "markdown")

|  | SA | NZ | Tas | Nat | ASe |
| --- | --- | --- | --- | --- | --- |
| SA | NA | 0.139 | 0.079 | 0.129 | 0.851 |
| NZ | 0.208 | NA | 0.188 | 0.020 | 0.693 |
| Tas | 0.010 | 0.010 | NA | 0.030 | 1.000 |
| Nat | 0.218 | 0.129 | 0.050 | NA | 0.198 |
| ASe | 0.861 | 0.762 | 1.000 | 0.079 | NA |

Niche Stability null model (p-values):

kable(sim_stability, digits = 3, format = "markdown")

|  | SA | NZ | Tas | Nat | ASe |
| --- | --- | --- | --- | --- | --- |
| SA | NA | 0.139 | 0.079 | 0.129 | 0.851 |
| NZ | 0.208 | NA | 0.188 | 0.020 | 0.693 |
| Tas | 0.010 | 0.010 | NA | 0.030 | 1.000 |
| Nat | 0.218 | 0.129 | 0.050 | NA | 0.198 |
| ASe | 0.861 | 0.762 | 1.000 | 0.079 | NA |

##

## **Figure results**

**Individual niche plots**

We developed some modifications in the plot.niche function available at Broennimann et al. 2012. The modifications include more options and flexibility to the plot.

plot.niche.mod <- function(z, name.axis1 = "PC1", name.axis2 = "PC2",
 cor = F, corte, contornar = TRUE,
 densidade = TRUE, quantis = 10,
 back = TRUE, x = "red", title = "",
 i) {
 cor1 <- function(cores.i, n) {
 al <- seq(0,1,(1/n))
 cores <- numeric(length(n))
 for(i in 1:n) {
 corespar <- col2rgb(cores.i)/255
 cores[i] <- rgb(corespar[1, ], corespar[2, ],
 corespar[3, ], alpha = al[i])
 }
 return(cores)
 }
 a1 <- colorRampPalette(c("transparent",cor1(x, quantis)), alpha = TRUE)
 xlim <- c(min(sapply(z, function(x){min(x$x)})),
 max(sapply(z, function(x){max(x$x)})))
 ylim <- c(min(sapply(z, function(x){min(x$y)})),
 max(sapply(z, function(x){max(x$y)})))
 graphics::image(z[[1]]$x, z[[1]]$y,
 t(as.matrix(z[[i]]$z.uncor))[, nrow(as.matrix(z[[i]]$z.uncor)):1],
 col = "white",
 ylim = ylim, xlim = xlim,
 zlim = c(0.000001, max(as.matrix(z[[1]]$z.uncor), na.rm = T)),
 xlab = "PC1", ylab = "PC2", cex.lab = 1.5,
 cex.axis = 1.4)
 abline(h = 0, v = 0, lty = 2)
 if (back) {
 contour(z[[i]]$x, z[[i]]$y,
 t(as.matrix(z[[i]]$Z))[, nrow(as.matrix(z[[i]]$Z)):1],
 add = TRUE, levels = quantile(z[[i]]$Z[z[[i]]$Z > 0],
 c(0, 0.5)), drawlabels = FALSE,
 lty = c(1, 2), col = x, lwd = 1)
 }
 if (densidade) {
 image(z[[i]]$x, z[[i]]$y, t(as.matrix(z[[i]]$z.uncor))[, nrow(as.matrix(z[[i]]$z.uncor)):1], col = a1(100), add = TRUE)
 }
 if(contornar){
 contour(z[[i]]$x, z[[i]]$y, t(as.matrix(z[[i]]$z.uncor))[, nrow(as.matrix(z[[i]]$z.uncor)):1],
 add = TRUE, levels = quantile(z[[i]]$z.uncor[z[[i]]$z.uncor > 0],
 seq(0, 1, (1 / quantis))),
 drawlabels = FALSE, lty = c(rep(2,(quantis - 1)), 1),
 col = cor1(x, quantis), lwd = c(rep(1, (quantis - 1)), 2))
 }
 title(title)
 box()
}

We applied this function here to plot all individual results per group. The continuous line represents 100% of the available environmental background, and the dashed line represents 50% of the most common conditions.

for(i in 1:n.groups) {
 plot.niche.mod(z, name.axis1 = "PC1", name.axis2 = "PC2",
 cor = F, corte, contornar = FALSE,
 densidade = TRUE, quantis = 10,
 back = TRUE, x = g.colors[i], title = g.names[i], i)
}

**Multiple niche plots**

We also modified the same function to allow multiple regions/species plots.

plot.niche.all <- function(z, n.groups, g.names,
 contornar = TRUE,
 densidade = TRUE,
 quantis = 10,
 back = TRUE, title = "",
 g.colors, n = 5,
 cor1) {
 # Color func
 cor1 <- function(cores.i, n) {
 al <- seq(0,1,(1/n))
 cores <- numeric(length(n))
 for(i in 1:n) {
 corespar <- col2rgb(cores.i)/255
 cores[i] <- rgb(corespar[1, ], corespar[2, ],
 corespar[3, ], alpha = al[i])
 }
 return(cores)
 }
 a <- list()
 for(i in 1:n.groups) {
 a[[i]] <- colorRampPalette(c("transparent", cor1(g.colors[i], n)),
 alpha = TRUE)
 }
 xlim <- c(min(sapply(z, function(x){min(x$x)})),
 max(sapply(z, function(x){max(x$x)})))
 ylim <- c(min(sapply(z, function(x){min(x$y)})),
 max(sapply(z, function(x){max(x$y)})))
 image(z[[1]]$x, z[[1]]$y, t(as.matrix(z[[1]]$z.uncor))[, nrow(as.matrix(z[[1]]$z.uncor)):1], col = "white",
 ylim = ylim, xlim = xlim,
 zlim = c(0.000001, max(as.matrix(z[[1]]$Z), na.rm = T)),
 xlab = "PC1", ylab = "PC2", cex.lab = 1.5,
 cex.axis = 1.4)
 abline(h = 0, v = 0, lty = 3)
 box()
 if (back) {
 for(i in 1:n.groups) {
 contour(z[[i]]$x, z[[i]]$y, t(as.matrix(z[[i]]$Z))[, nrow(as.matrix(z[[i]]$Z)):1], add = TRUE,
 levels = quantile(z[[i]]$Z[z[[i]]$Z > 0], c(0, 1)),
 drawlabels = FALSE,lty = c(2),
 col = g.colors[i], lwd = 1)
 }
 }
 if (densidade) {
 for(i in 1:n.groups) {
 image(z[[i]]$x, z[[i]]$y, t(as.matrix(z[[i]]$z.uncor))[, nrow(as.matrix(z[[i]]$z.uncor)):1],
 col = a[[i]](100), add = TRUE)
 }
 }
 if(contornar){
 for(i in 1:n.groups) {
 contour(z[[i]]$x, z[[i]]$y, t(as.matrix(z[[i]]$z.uncor))[, nrow(as.matrix(z[[i]]$z.uncor)):1], add = TRUE,
 levels = quantile(z[[i]]$z.uncor[z[[i]]$z.uncor > 0],
 seq(0, 1, (1/quantis)))[quantis],
 drawlabels = FALSE, lty = rev(c(rep(2,(quantis - 1)), 1)),
 col = rev(cor1(g.colors[i], quantis)),
 lwd = rev(c(rep(1, (quantis - 1)), 2)))
 }
 }
}

The results can be seen here. The strong contours represent the 20% highest values of density, and the dashed thin lines represent 100% of the background available in each region.

plot.niche.all(z, n.groups, g.names,
 contornar = TRUE,
 densidade = TRUE,
 quantis = 4,
 back = FALSE, title = "",
 g.colors, n = 3,
 cor1)
plot.niche.all(z, n.groups, g.names,
 contornar = TRUE,
 densidade = TRUE,
 quantis = 4,
 back = TRUE, title = "",
 g.colors, n = 3,
 cor1)

Regular plot:

col1 <- colorRampPalette(c(desaturate(g.colors[1]), g.colors[1]))(5)
col2 <- colorRampPalette(c(desaturate(g.colors[2]), g.colors[2]))(5)
col3 <- colorRampPalette(c(desaturate(g.colors[3]), g.colors[3]))(5)
col_int <- colorRampPalette(c(desaturate('#e7298a'), '#e7298a'))(5)
for (i in 2:n.groups) {
 ecospat.plot.niche.dyn(z[[1]], z[[i]], .5,
 colZ1 = g.colors[1],
 colZ2 = g.colors[i],
 col.stab = "gray",
 col.unf = g.colors[1],
 col.exp = g.colors[i],
 name.axis1 = "PC1",
 name.axis2 = "PC2",
 transparency = 70)
 mtext(paste(species[1],"x", species[i]), font = 3)
 }


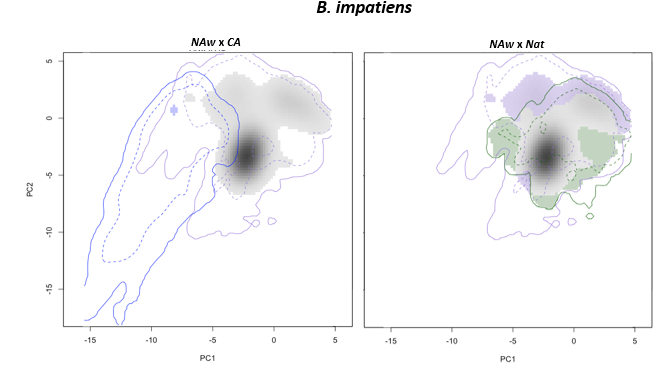


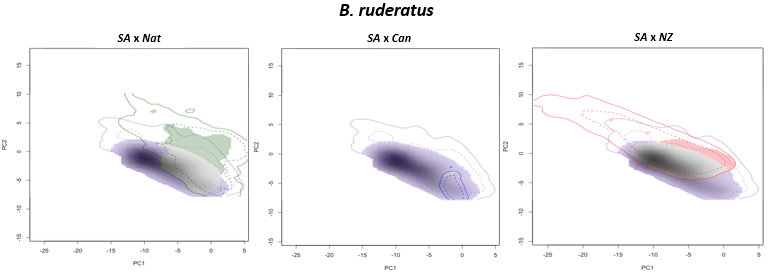


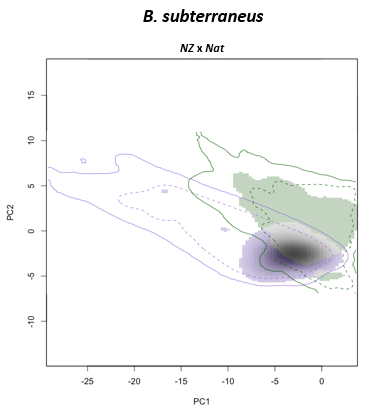


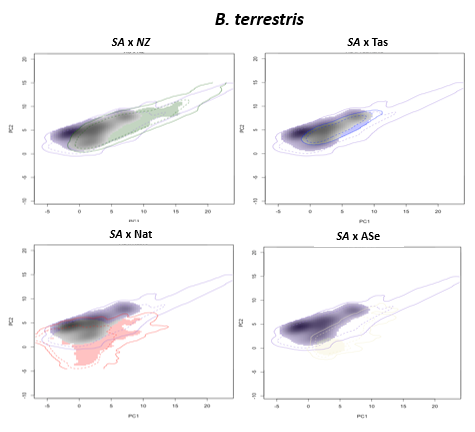


Below is the loadings plot (contribution of the variables for each axis). Check the variable codes at <http://www.worldclim.org/bioclim>.

loadings <- cbind(cor(data.env, pca.cal$tab[,1]), cor(data.env, pca.cal$tab[,2]))
colnames(loadings) <- c("axis1", "axis2")
loadings <- loadings[c(1, 12:19, 2:11), ]
barplot(loadings[,1], las=2, main="PC1")
barplot(loadings[,2], las=2, main="PC2")

The arrows represent the contribution of each variable directly to the environmental space.

contrib <- pca.cal$co
eigen <- pca.cal$eig
nomes <- numeric(19)
for(i in 1:19){
 nomes[i] <- paste('bio',i, sep="")
}
s.corcircle(contrib[, 1:2] / max(abs(contrib[, 1:2])),
 grid = F, label = nomes, clabel = 1.2)
text(0, -1.1, paste("PC1 (", round(eigen[1]/sum(eigen)*100,2),"%)",
 sep =””))
text(1.1, 0, paste("PC2 (", round(eigen[2]/sum(eigen)*100,2),"%)",
 sep = ""), srt = 90)

## References

Aiello‐Lammens, M. E., Boria, R. A., Radosavljevic, A., Vilela, B., & Anderson, R. P. (2015). spThin: an R package for spatial thinning of species occurrence records for use in ecological niche models. *Ecography,* 38(5), 541-545.

Andrade, A. F. A., Velazco, S. J. E., & De Marco Júnior, P. (2020). ENMTML: An R package for a straightforward construction of complex ecological niche models. *Environmental Modelling and Software*, 125, 1-10. https://doi.org/10.1016/j.envsoft.2019.104615

Broennimann, O., Fitzpatrick, M. C., Pearman, P. B., Petitpierre, B., Pellissier, L., Yoccoz, N. G., & Guisan, A. (2012). Measuring ecological niche overlap from occurrence and spatial environmental data. *Global Ecology and Biogeography*, 21(4), 481-497.
